# Fatty Acid Composition of Selected Street Foods Commonly Available in Malaysia

**DOI:** 10.3390/foods12061234

**Published:** 2023-03-14

**Authors:** Zainorain Natasha Zainal Arifen, Mohd Razif Shahril, Suzana Shahar, Hamdan Mohamad, Siti Farrah Zaidah Mohd Yazid, Viola Michael, Tanaka Taketo, Kathy Trieu, Sakinah Harith, Nor Hayati Ibrahim, Shariza Abdul Razak, Hanapi Mat Jusoh, Chua Hun Pin, Jau-Shya Lee, Risyawati Mohamed Ismail, Lee Lai Kuan, Hasnah Haron

**Affiliations:** 1 Nutritional Sciences Programme, Centre for Healthy Ageing and Wellness (H-Care), Faculty of Health Sciences, Universiti Kebangsaan Malaysia, Kuala Lumpur 50300, Malaysia; zainorainatasha@gmail.com (Z.N.Z.A.);; 2 Dietetic Programme, Centre for Healthy Ageing and Wellness (H-Care), Faculty of Health Sciences, Universiti Kebangsaan Malaysia, Kuala Lumpur 50300, Malaysia; 3 Non-Communicable Disease Section, Disease Control Division, Ministry of Health, Putrajaya 62590, Malaysia; 4 Enforcement Section, Allied Health Sciences Division, Ministry of Health, Putrajaya 62050, Malaysia; 5 Representative Office for Malaysia, Brunei Darussalam, and Singapore, World Health Organization, Cyberjaya 63000, Malaysia; 6 The George Institute for Global Health Level 5, 1 King St, Newtown, NSW 2042, Australia; 7 Nutrition & Dietetic Programme, Faculty of Health Sciences, Universiti Sultan Zainal Abidin, Kuala Nerus 21300, Malaysia; 8 Faculty of Fisheries and Food Science, Universiti Malaysia Terengganu, Kuala Nerus 21030, Malaysia; 9 Nutrition Programme, School of Health Sciences, Health Campus, Universiti Sains Malaysia, Kubang Kerian 16150, Malaysia; 10 Department of Nutrition Sciences, Kulliyyah of Allied Health Sciences, International Islamic University Malaysia, Kuantan 25200, Malaysia; 11 Food Science and Technology Research Centre, Malaysia Agricultural Research and Development Institute, Kuching 93050, Malaysia; 12 Faculty of Food Science and Nutrition, Universiti Malaysia Sabah, Kota Kinabalu 88400, Malaysia; 13 School of Technology Management & Logistics, Universiti Utara Malaysia, Sintok 06010, Malaysia; 14 Food Technology Programme, School of Industrial Technology, Universiti Sains Malaysia, Gelugor 11800, Malaysia

**Keywords:** street food, fatty acid composition, saturated fat, trans fat, preparation, Malaysia, deep-frying, coconut milk, processed food

## Abstract

Despite growing evidence of increased saturated and trans fat contents in street foods, little is known about their fatty acid (FA) compositions. This study aimed to analyse the saturated fatty acids (SFAs), monounsaturated fatty acids (MUFAs), polyunsaturated fatty acids (PUFAs), and trans fatty acids (TFAs) content of 70 selected and most commonly available street foods in Malaysia. The street foods were categorised into main meals, snacks, and desserts. TFAs were not detected in any of the street foods. Descriptively, all three categories mainly contained SFAs, followed by MUFAs, and PUFAs. However, the one-way ANOVA testing showed that the differences between each category were insignificant (*p* > 0.05), and each FA was not significantly different (*p* > 0.05) from one to another. Nearly half of the deep-fried street foods contained medium to high SFAs content (1.7 g/100 g–24.3 g/100 g), while the MUFAs were also high (32.0–44.4%). The Chi-square test of association showed that the type of preparation methods (low or high fat) used was significantly associated (*p* < 0.05) with the number of SFAs. These findings provide valuable information about fat composition in local street foods for the Malaysian Food Composition Database and highlight the urgency to improve nutritional composition.

## 1. Introduction

Globalisation has resulted in a nutrition transition towards high-fat, sugar, and salty foods in low- and middle-income countries (LMICs), including Malaysia, an upper-middle-income country [1]. Street foods are easily accessible [2], widely available, and inexpensive compared to other formal food premises [3]; hence, they are a major contributor to dietary intake and nutrition for most of the population. Fats in food exist in varying proportions of saturated fatty acids (SFAs), monounsaturated fatty acids (MUFAs), polyunsaturated fatty acids (PUFAs) and trans fatty acids (TFAs) [4]. Several studies from other countries (Eastern Europe, Central Asia, Southeast Asia and Africa) found the presence of TFAs [5,6,7,8,9,10,11] and high amounts of SFAs [6,7,8] in their local street food.

The presence of TFAs and SFAs may be influenced by the use of TFAs- or SFAs-rich ingredients, such as butter [7] and partially hydrogenated vegetable oils [9,12], and the preparation methods used, such as deep-frying [9,11,13]. Practices related to the frying process, such as the reuse of frying oil [6], the frying temperature [14], and the duration of frying using different types of oils [10,13,15,16,17], also affect the levels of SFAs and TFAs in foods. As most Malaysian foods incorporate ingredients that are rich in fats, such as coconut milk and peanuts, and approximately 30% of Malaysian street foods are deep-fried [18], local street foods may contain high amounts of SFAs and TFAs, as well as MUFAs and PUFAs [13].

Knowledge of the fatty acids (FAs) composition in foods is crucial because the high consumption of SFAs [19,20] and TFAs [21] has been associated with adverse health effects, including hyperlipidaemia and hypercholesterolemia. Meanwhile, the consumption of unsaturated fats is associated with many health benefits. A meta-analysis [22] concluded that unsaturated fatty-rich oils were more effective in lowering low-density lipoprotein-cholesterols (LDL-C) than highly saturated solid fats. Although there was inconsistent evidence to associate SFAs intake with cardiovascular diseases (CVDs), healthy dietary patterns that reduce CVD risk are typically not high in SFAs content [23]. In fact, replacing SFAs with PUFAs is associated with CVD risk reduction [24]. Given that CVD deaths are the leading cause of death worldwide and are responsible for one in three deaths in Malaysia [25], knowledge about fat levels in foods could inform food and nutrition policies for preventing CVD.

Documentation of FA composition in Malaysian street foods is still lacking compared to the well-documented FA composition in street foods from other countries [5,6,7,8,9,10,11]. Previous studies in Malaysia have measured the FA composition in homemade meals [26] and the presence of TFAs in foods ranging from bakery products, snacks, breakfast cereals, dairy products, fast foods, Malaysian fast foods [27] to supermarket foods [28], but did not assess the FAs levels in street foods. Currently, only 23 ready-to-eat foods have their FA composition available in the Malaysian Food Composition Database (MyFCD) [29]. Therefore, this study aimed to analyse the FA composition of the commonly available street foods in Malaysia. Data from this study would expand the FA composition database in the MyFCD, which is used to provide consumers with public information on FA levels in foods and offer better quality nutrient data, especially for those involved in food preparation [30]. The detailed abbreviations and definitions used in the paper are listed in Table 1.

## 2. Materials and Methods

In this cross-sectional study, data collection was conducted in two phases: (1) a survey of street foods and (2) sampling and analysis of FA composition in selected street foods. In Phase 1 of the study, a survey was conducted in all states to identify the top 15 most frequently available street foods for each food category in every state. In Phase 2 of the study, food sampling of 70 street foods (5 foods × 14 states) was carried out to analyse the FA composition. However, among the 70 analysed street foods, 14 similar foods were sampled from more than one state, and 25 different foods were sampled from only one state. In order to achieve the aim of this study, the FA contents of the 14 similar foods were presented as average values from the respective states. Thus, this study presented the FA composition for 39 street foods. 

This study is an extension of previous research undertaken to determine the most frequently available street food in all states of Malaysia while assessing the sodium levels [18]. Furthermore, the current study applied an additional sampling criterion for FAs composition analysis in Phase 2. This study was carried out from October 2021–May 2022. The methods of conducting this study have been approved by the Research Ethics Committee of the National University of Malaysia with reference number UKMPPI/111/8/JEP-2020-433.

### 2.1. Phase 1: Survey of Street Food in All States of Malaysia

Phase 1 involved a field survey of locally available street foods in 13 states and one Federal Territory of Kuala Lumpur in Malaysia. The definition of street food was adopted from the Food and Agriculture Organization (FAO) [31]: “Ready-to-eat foods consumed without further processing or preparation, and sold by roadside hawkers such as trolleys, bicycles, markets, trucks or stalls that do not have fixed building or four walls.” Therefore, the eligible street food vending sites included in the survey were food establishments selling ready-to-eat food and not contained within a fixed building or four walls, i.e., mobile and stationary vending sites, individual stalls, and stalls at the morning and night markets. The surveyed markets were identified through the city council websites of each state. The study had a total of 380 locations surveyed across 68 districts in Malaysia. 

A survey form was used to record information such as the name of street food, state, district, category of street food (e.g., main meal, snack, or dessert), and preparation method of the street food. The criteria for main meals, snacks, and desserts in this study are based on the description by the International Scientific Committee [32] as follows:

1. Main meal: food commonly eaten during main mealtimes, i.e., breakfast, lunch, and dinner;
2. Snack: savoury food eaten between the main mealtimes, i.e., morning tea and afternoon tea;
3. Dessert: sweet food eaten at the end of a main meal or as part of the main meal.

A total of 10,520 street foods were surveyed, in which 40% (*n* = 4234) of the street foods were snacks, while 37% (*n* = 3887) and 23% (*n* = 2399) were the main meals and dessert categories, respectively. The top 15 most frequently available street foods for each category in all states were subsequently identified and recorded.

### 2.2. Phase 2: Sampling and Analysis of FA Composition in Selected Street Foods

#### 2.2.1. Sampling of Street Food from Each State

The selection of food for sampling in each state was conducted based on the top 15 most frequently available street foods for each category previously identified in Phase 1 (in which several types of street foods varied between states) as well as the following criteria: (1) use of high-fat preparation method, (2) use of high-fat ingredients, and (3) availability of the street food during the sampling (food sampling was conducted during the COVID-19 pandemic; hence the operating hours of stalls were hugely affected). Based on the selection, five street foods (regardless of the food categories) from every state were selected for food sampling, according to a method used by Tee et al. [33]. Thus, 70 street food samples from 14 states (including the same type of food from a different state) were analysed for the FA composition. While sampling each selected food, samples were purchased from two stalls within the respective states. This also applied to foods that were sampled from only one state. For instance, *kerepek* (only found in one state) was analysed based on two *kerepek* purchases from stall 1 and stall 2 within the respective state. The purchased food samples were transported in an ice box and stored in the freezer at −20 °C to delay food spoilage before further analysis.

#### 2.2.2. Preparation of Street Food Samples for FA Composition Analysis

The preparation of street food samples was conducted in the food analysis laboratory. Each purchased food sample was weighed with the packaging using a top pan balance (Mettler Toledo, OH, USA, Dragon 3002). Then, the food was removed from the packaging, weighed, and placed on a plate or bowl. The inedible portions of the food samples, such as bones, were removed, and the sample was reweighed. The second sample of the same food purchased from a different location was also prepared using the same method. The two samples were then homogenised in a food processor, scooped into an airtight container, and subsequently stored in the freezer at −20 °C for further analysis. Prior to the FA composition analysis, the determination of fat content using the homogenised samples was first carried out through the Soxhlet method [34]. The extraction, esterification, and analysis of FAs conducted in duplicates of the homogenised samples were performed within the same day. 

#### 2.2.3. Chemicals and Solvents

Methanol (99.9%), chloroform, potassium chloride (0.88%), and sodium sulphate were used during the extraction of FAs. Meanwhile, toluene, 2% sulphuric acid in methanol, sodium chloride (5%), heptane, and sodium bicarbonate (2%) were used during the esterification of FAs. The Reference Standard 37 Component FAME Mix (Supelco, St. Louis, MO, USA, 47885-U, 10 mg/mL) was used to analyse FAs using the Gas chromatography flame ionization detection (GC-FID) method. All reagents and standards were prepared with deionised water, except for the 2% sulphuric acid in methanol (2 mL in 100 mL methanol).

#### 2.2.4. Extraction of FAs

Approximately 1.5 g (±0.1 g) of the homogenised sample was placed into a glass bottle. Next, 10 mL of methanol and 20 mL of chloroform were added to the glass bottle. The glass bottle was then vortexed and left to sediment for a few minutes. The solvent extracts were then filtered into a Schott bottle by leaving a small portion of the sample in the glass bottle to retain any food debris. The extraction process was repeated an additional two times. After the extraction, 20 mL of 0.88% potassium chloride was added to the Schott bottle. The bottle was then shaken to allow the separation into two layers, and the upper layer was discarded. Similarly, 10 mL of 0.88% potassium chloride and 10 mL methanol mixture (ratio 1:1) were added into the same Schott bottle and shaken to allow separation, and the upper layer was discarded. The solvent extract was filtered through sodium sulphate and poured into a new glass bottle to remove moisture. The extracts were then concentrated under nitrogen gas below 60 °C.

#### 2.2.5. Esterification of FAs

The extract containing the FAs underwent an esterification process to form fatty acid methyl esters (FAMEs). During this procedure, the sample dried with nitrogen gas was mixed with 1 mL of toluene and 2 mL of 2% sulphuric acid in methanol in the same glass bottle. The glass bottle was incubated overnight at 50–55 °C. After incubation, the following steps were conducted and repeated twice: (1) 5 mL of 5% sodium chloride in water was added to the glass bottle, and (2) 5 mL of heptane was added, shaken slightly, and left for a few minutes. The upper layer (heptane) was then transferred into a new glass bottle and combined with the subsequent upper layers (heptane) from the repeated process. Then, 4 mL of 2% sodium bicarbonate in water was added to the extract. The sample mixture was shaken and left to separate. The upper layer was filtered through sodium sulphate and placed in a new glass bottle. The 1 mL of the filtered n-heptane layer was pipetted from the glass bottle directly into the vials. Then, a 10× dilution using n-heptane was performed by mixing 100 μL of the sample and 900 μL of n-heptane in a 2 mL vial.

#### 2.2.6. Detection and Quantitation of FAs by GC-FID

Blanks for each batch of samples were run through the entire procedure to measure any contributions to residue from reagents. GC-FID analysis was carried out using a gas chromatograph (Agilent 6890, Santa Clara, CA, USA) equipped with a ZB-FAME GC Column (60 m × 0.25 mm × 0.2 µm) and a flame ionisation detector (FID). The chromatographic conditions were as follows: initial column temperature 100 °C; injection temperature 250 °C; detector temperature 280 °C; and run time 64 min. The flow rate of the carrier gas (nitrogen) was set at 20 mL/min.

In this method, 1 μL of the methyl esters (extract) solution was injected with the reference standard mixture into the GC-FID. The reference standard mixture was analysed under the same operating conditions as those employed for the sample, and the retention times were measured, along with the eluted methyl ester from the reference standard mixture were identified. The limit of detection (LOD) for all FAs was 0.01 mg/100 g.

The quantification and identification of FAs present in the food sample were conducted using the peak area normalization method. Graphs showing the logarithm of the retention distance of each peak were constructed. All peaks in the sample chromatogram (including those that were not within the 37 FAME retention time) were integrated. The entire sample component was assumed to be represented on the chromatogram. Hence, the total area under the peaks represents 100% of the constituents. As for the result, only 37 components were reported based on the reference standards.

The analytical results of SFAs, MUFAs, PUFAs, and TFAs in each sample were expressed as a percentage (%) based on total fats in the respective samples, similar to the MyFCD. For the purpose of classifying the street food samples based on the low, medium, or high content of SFAs, the amount of SFAs was also expressed as g/100 g of food. The percentage of SFAs based on total FAs was converted into grams of SFAs based on 100 g of food using the following calculation:

(1) $$\mathrm{SFA}(g/100\;g\;\mathrm{food})=\frac{Fat\;\left(\frac{g}{100}g\;food\right)\times SFA\;\left(\%\;total\;fat\right)}{100\%}$$

#### 2.2.7. Classification of SFAs Content

There is no standardised classification for high SFAs, MUFAs, PUFAs, and TFAs in ready-to-eat dishes like street foods in Malaysia. However, the United Kingdom’s Traffic Light Labelling Scheme [35] has a classification for labelling low (≤1.5 g/100 g food), medium (>1.5 g to ≤5 g/100 g food), and high (>5 g/100 g food) SFAs in foods. Thus, this study adopted this classification for the SFAs content of street foods. 

#### 2.2.8. Classification of Preparation Method

Based on the latest Malaysian Dietary Guidelines (MDG) [36], the preparation methods identified in the survey were further classified as either healthier (e.g., pan-frying, steaming, stir-frying, grilling, stewing, and baking) or less healthy (e.g., deep-frying), to identify whether the preparation methods affected the SFAs content of street foods.

### 2.3. Statistical Analysis

A descriptive test was used to determine the total frequency of every street food surveyed for each state, the frequency of the street foods based on food categories, the preparation methods, and the type of street foods. Following the analysis of FA composition, a descriptive test was also used to determine the average and standard deviation of each FA for the selected street foods. Inferential test such as the one-way ANOVA was used to compare the average SFAs, MUFAs, and PUFAs between food categories. If the food categories had unequal sample sizes and both one-way ANOVA and homogeneity of variance were significant, a Games-Howell posthoc test was performed to identify the specific differences between the three food categories. The one-way ANOVA was also used to compare each FAs within each food category. The Chi-square test of association was used to determine the association between the SFAs content (g/100 g food) and the preparation method used for each street food. All the descriptive and statistical tests were conducted using IBM (Armonk, NY, USA) Statistical Package for Social Sciences (SPSS) version 25.0. The significance level for all conducted statistical analyses was set at *p* < 0.05.

## 3. Results

A total of 70 street foods (32 snacks, 26 main meals, and 12 desserts) were analysed for their FA composition, as displayed in Table S1. This paper presents the SFAs, MUFAs, PUFAs, and TFAs contents of similar street foods from different states as average values. Since 25 street foods were obtained from only one state, and 14 similar street foods were obtained from more than one state, this study reported the average SFAs, MUFAs, PUFAs, and TFAs contents in 39 selected street foods (16 snacks, 14 main meals, and 9 desserts) that are frequently available in Malaysia. The description of each street food is shown in Table S2. 

**Figure 1:**
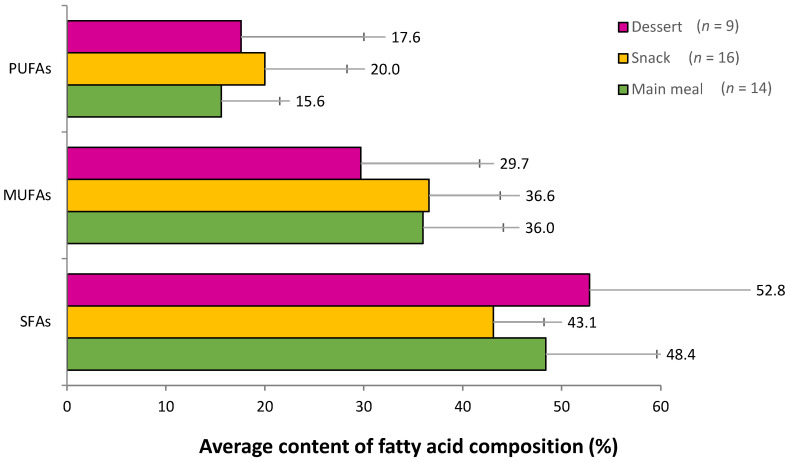
The average fatty acid (FA) composition from 39 street foods based on food category. PUFAs, MUFAs, and SFAs are the abbreviations for polyunsaturated fatty acids, monounsaturated fatty acids, and saturated fatty acids, respectively.

None of the analysed street foods contained any amount of TFAs. Figure 1 shows the average content of SFAs, MUFAs, and PUFAs based on the food category (16 snacks, 14 main meals, and 9 desserts, respectively). The dessert category had the highest SFAs content (52.8 ± 18.2%), followed by the main meals (48.4 ± 11.2%) and snacks (43.1 ± 5.1%). MUFAs content was the highest in snacks (36.6 ± 7.2%), followed by main meals (36.0 ± 8.1%), and desserts (29.7 ± 12.0%). PUFAs content was the highest in snacks (20.0 ± 8.3%), followed by desserts (17.6 ± 12.4%) and main meals (15.6 ± 5.9%). The FAs content between the food categories was not significantly different (*p* > 0.05) from one to another. Within each food category, all three food categories were composed mainly of SFAs, followed by MUFAs, and the least was in PUFAs, although the findings were not significantly different (*p* > 0.05). 

Table 2 shows the preparation method and the average FA composition of the 39 street foods arranged according to the classification of high, medium, and low SFAs content per 100 g of food. In general, the SFAs, MUFAs, and PUFAs content of street foods ranged from 29.3–88.5%, 7.0–44.4%, and 4.5–38.8%, respectively.

Based on the classification of SFAs content in 100 g of food sample, *cakoi* (24.3 ± 3.6 g/100 g) contained the highest SFAs content, while chicken rice (1.2 ± 0.3 g/100 g) had the lowest SFAs content. 

Most of the analysed main meals (85.7%) and all the snacks and desserts contained medium to high SFAs content, ranging from 1.7–24.3 g/100 g. According to the classification [35], 56.4% of the street foods had medium SFAs content (1.7–4.5 g/100 g), while the other 38.5% had high SFAs content (5.1–24.3 g/100 g). Among the foods with medium to high SFAs contents, 43.2% were deep-fried, which included *cakoi*, *kerepek*, french fries, chicken nuggets, and donuts. This was followed by other preparation methods, such as pan-frying (21.6%), steaming (13.5%), stir-frying (8.1%), grilling (8.1%), stewing (2.7%), and baking (2.7%). Meanwhile, only 5.1% of the 39 street foods contained low SFAs content (1.2–1.4 g/100 g). The foods with low SFAs content were those prepared by stir-frying (fried vermicelli) and steaming (chicken rice).

There were 32 street foods that contained the highest MUFAs content (32.0–44.4%), mostly found in snacks (43.8%), main meals (40.6%), and desserts (15.6%). Compared to the other street foods, fried vermicelli contained the most MUFAs, while *kuih jelurut* had the least. The majority (46.9%) of the street foods were prepared using deep-fried methods such as curry puff, fried chicken with cheese, banana fritters, *kuih keria*, *kerepek*, fried *popiah*, chicken nuggets, fried fish ball and donuts. This was followed by street foods that were pan-fried (21.9%), stir-fried (12.5%), steamed (9.4%), grilled (6.3%), and stewed (3.1%). The remaining 7 street foods contained MUFAs ranging from 7.0% to 27.5%. The MUFAs content for all 39 street foods, from highest to lowest amount, is shown in Table S3.

Surprisingly, only 7 street foods had the highest amount of PUFAs (30.0–38.8%), mainly found in snacks (57.1%), desserts (28.6%), and main meals (14.3%). Approximately 42.9% were pan-fried, 28.6% were grilled (28.6%), and 14.3% were deep-fried and baked, respectively. The remaining 32 street foods contained PUFAs ranging from 4.5% to 24.6%. *Apam balik* was reported to contain the highest amount of PUFAs, while *kuih jelurut* had the least amount of PUFAs. The PUFAs content for all 39 street foods, from highest to lowest amount, is shown in Table S4.

**Figure 2:**
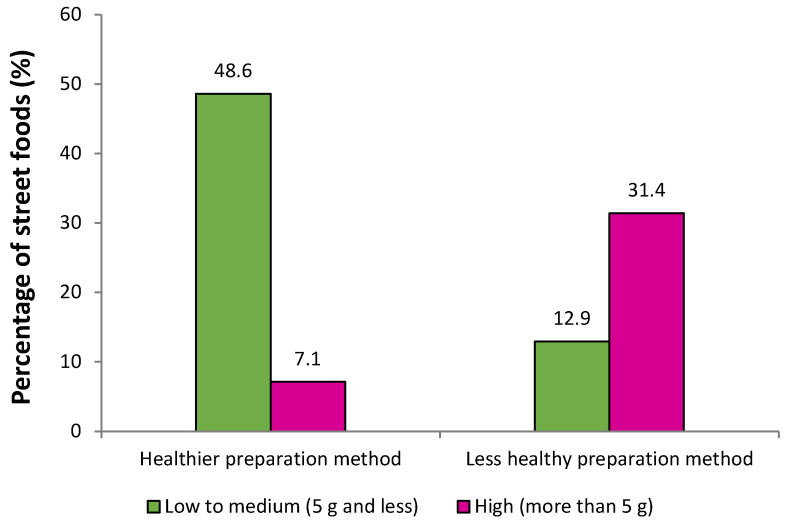
Percentage of street foods (*n* = 70) based on the classification of preparation methods and SFAs content in 100 g of food.

Figure 2 displays the percentage distribution of all 70 street foods based on the classifications of the preparation methods and SFAs content. Most (48.6%) of the street foods that were prepared using healthier preparation methods, such as steaming, baking, stewing, grilling, pan-frying, and stir-frying, contained low to medium SFAs content. This was followed by street foods that used a less healthy preparation method (i.e., deep-frying) and contained high (31.4%) and low to medium (12.9%) SFAs content, respectively. Lastly, only 7.1% of street foods that were prepared in a healthier manner reported a high SFAs content of more than 5 g/100 g of food. An association test (shown in Table 3) reported that the SFAs content in the 70 street foods was significant (*p* < 0.001) and strongly associated (Φ = 0.59) with the classification of the preparation methods used. This means that foods prepared using a healthier method were more likely to contain low to medium SFAs contents than foods prepared less healthily.

## 4. Discussion

This study found that the gas chromatography did not detect TFAs in any of the local street foods, which is not in agreement with the findings from other countries in the Asian [5,6,8,12], European [7,9,37], and African [10] regions. This is possibly due to varying ingredients used in the local street foods in other countries. For instance, street foods in other Asian countries, such as India [12], are mainly fried in or used *vanaspati*, a vegetable ghee rich in TFAs [38] that is not commonly used in Malaysian street food. Despite the fact that a large proportion of street foods in the current study are also deep-fried, it is possible that palm oil was used by the local vendors, as it is a common cooking or frying oil in Malaysia [39]. According to the Malaysian Palm Oil Council [40], palm oil is popularly used in the local food scene as it is naturally stable against oxidation when used for high-temperature cooking methods, such as deep-frying, due to the high composition of SFAs in the oil. Moreover, the semi-solid form enables palm oil to be used without the need to be further solidified through the process of hydrogenation, making palm oil free from TFAs [41]. Besides *vanaspati*, other sources of TFAs, such as partially hydrogenated margarine and shortenings, are commonly used in street foods, such as *roti canai*, curry puff, chicken burgers, and donuts [36]. However, the findings in this study may suggest that the use of partially hydrogenated ingredients among vendors in Malaysia is low and that they might use tub-style margarine products which do not contain TFAs [42]. Notably, the substitution of stick margarine with tub margarine was found to be associated with a lower risk of CVD [43]. Other than the type of fat-based ingredients used in the local street foods, the absence of TFAs may also be explained by the cooking temperature used to prepare the foods. Most of the studied street foods are deep-fried using a cooking temperature below 200 °C [44]. A recent review [14] found that heating edible oils to below 200 °C, a common cooking temperature, does not affect the TFAs levels in the oil. Yi Chen et al. [17] also found that there was only a small increase in TFAs levels in foods deep-fried below 200 °C using palm oil. 

In this study, desserts contained the most SFAs, whereas snacks had the most MUFAs and PUFAs. These findings were in line with street food studies in Kazakhstan [5] and Moldova [7], in which SFAs content was significantly the highest in street foods that are sweet, such as chocolates, cakes, and wafers. Albuquerque et al. [7] also reported that PUFAs were highest in savoury street foods, such as *pateuri* (a fried pastry). In this study, all street foods under the dessert category were sweet; meanwhile, all street foods under the snack category were of the savoury type. Despite SFAs being the highest in desserts and MUFAs and PUFAs being the highest in snacks, SFAs were the most dominant FA present in all three food categories. This finding supports the evidence from a study [45] reporting that local Asian foods are, indeed, high in saturated fats and more saturated than westernised-styled foods. Although there is an ongoing debate on the link between saturated fats and CVD risk [46,47,48], with growing evidence [49] of contrasting associations between different dietary sources of SFAs and the risk of CVD (rather than the amount of SFAs), it is still imperative for Malaysians to control their consumption of local street foods, as more than half (64.4%) of the population consumes at least one out-of-home meal daily [50], as well as to be conscious of the sources of SFAs consumed. Nevertheless, the FA profile of each food category presented a similar proportion of SFAs, MUFAs, and PUFAs. This was also previously observed in a study conducted among local street foods in Mozambique [10]. As street foods are relatively less expensive than regular brick-and-mortar food establishments [3,51,52], this may indicate that local street foods could be a good source of MUFAs and PUFAs, especially among the consumers in developing countries [53,54]. 

The SFAs, MUFAs, and PUFAs content between the street foods varied considerably, indicating possible differences in the preparation methods or ingredients used [55]. *Cakoi* ranked first among the 39 street foods in terms of SFAs content and is classified as a high-SFA food. *Cakoi* is a long, golden-brown deep-fried strip of dough that originated in China but is now a popular snack in Malaysia. On the contrary, chicken rice contains the least SFAs and is classified as a low-SFA food. This is most likely due to the difference in preparation methods, as *cakoi* is deep-fried, whereas steaming and roasting methods are mainly used to prepare the rice and chicken in the chicken rice. *Cakoi*, in the present study, is deep-fried in palm oil, which explains the high SFAs content, considering that palm oil has approximately 50% SFAs [56,57,58]. On the other hand, the SFAs content of the chicken in chicken rice may have been reduced after undergoing the roasting process. Alina et al. [59] found that the roasting method slightly reduces the SFAs content in chicken meat. Chicken rice, in this study, had marginally higher SFAs (43.6%) than the current reports in MyFCD (39.5%). Additionally, nearly half of the street foods with medium to high SFAs content that were deep-fried (such as *cakoi*, *kerepek*, french fries, chicken nuggets, and donuts) may also be linked to the use of palm oil. Thus, street food vendors should be encouraged to strain the excess oil off the fried food, and consumers should be reminded to control their consumption of deep-fried street foods, which is also in accordance with the MDG 2020 [36]. 

It is important to note that street foods not prepared by deep-frying also contain medium to high SFAs, possibly due to the usage of vegetable- and animal-based fats. In terms of ingredients, the main meals, such as net crepes (*roti jala*), glutinous rice with *rendang*, noodles with gravy, *nasi lemak*, and *nasi lemak* with fried chicken and desserts, such as *kuih seri muka* and *kuih jelurut*, are all coconut milk-based dishes. Traditionally, coconut milk is used extensively in Malaysian main dishes and desserts (except for *kuih jelurut*) [60], such as in the curry gravy served with net crepes (*roti jala*)*,* the *rendang* and glutinous rice, and the *pandan* custard layer of *kuih seri muka* [61]. The rice in *nasi lemak* is also cooked with coconut milk [62]. *Kuih jelurut,* a local steamed delicacy among the Brunei ethnicities [63] in Sabah, incorporates coconut milk as the main ingredient [64]. As the SFAs in coconut milk are present in high proportions (92.0%) [36], this may corroborate the current findings. Vendors could opt for substituting coconut milk with fresh milk in coconut milk-based desserts and main meals. Marina and NurulAzizah [65] found that custard pudding and green curry prepared using fresh milk had significantly lower fat content than those prepared using coconut milk and fresh coconut milk. Besides vegetable-based fat, such as coconut milk, animal-based fats, such as butter, processed meats, animal fats, and cheese, are used in these street foods. Popcorn was the second street food with the highest SFAs content. This may be because the popcorn samples were coated with a caramel butter sauce, and the latter is one of the primary sources of saturated fats in the diet [66]. Chicken patties, fried sausage, chicken nuggets, and pizza also contain processed meats high in saturated fats. In addition, the meat in *satay,* also known as meat skewers, is often skewered with meat fats to add more flavour. As expected, street foods with added cheese, such as fried chicken, banana fritters, and *apam balik* with cheese, had more SFAs than the regular versions.

The majority (82.1%) of the street foods in this study contained high MUFAs content. The MUFAs content is likely from the use of palm oil used to deep-fry most of the foods and other MUFA-rich ingredients. Although palm oil contains a high percentage of SFAs, it is also rich in MUFAs, with a nearly equal amount of SFAs [67,68]. Hence, this might have accounted for the high SFAs content in deep-fried snacks, such as *cakoi*, *kerepek*, french fries, chicken nuggets, fried *popiah*, curry puff, fried chicken, fried chicken with cheese, *keropok lekor*, fried sausage and fried fish ball, and deep-fried desserts, such as donuts, *kuih keria*, banana fritters with cheese, and banana fritters. Besides palm oil, nuts and seeds are also good sources of MUFAs, contributing almost 50% to the FA profile [39,67,68]. The MUFAs content in main meals, such as *roti canai*, might be due to the lentils gravy or *dhal*, as these two are commonly served together. In contrast, *nasi lemak* and *apam balik* might be good sources of MUFAs due to the use of peanuts that predominantly contain MUFAs [69]. Likewise, the *kerepek* or deep-fried chips in this study also contained peanuts, which contribute additional MUFAs to this deep-fried snack. Chicken burgers also contain high MUFAs content, which may be linked to the chicken patty used. Laskowski et al. [70] reported that meat products, including processed poultry products, contribute as much as 18% of MUFAs to the average Polish diet. Interestingly, noodle-based and rice-based main meals, such as fried *kuey teow*, fried noodles, noodles with gravy, fried rice, and chicken rice, were also among the high-MUFA-containing foods. The low MUFA (7%) content in *kuih jelurut* was possibly due to the lack of MUFA-rich ingredients used in this sweet dessert, as the main ingredients [71] (p. 148) needed are rice flour, *gula apong*, and coconut milk. The rich source of MUFAs reported in fried vermicelli should be made known to the consumers, as this main meal is one of the most commonly consumed ready-to-eat dishes among adults in Malaysia [72]. 

*Apam balik* and *apam balik* with cheese were the two street foods with the most amount of PUFAs. In a normal set of *apam balik*, this sweet pancake-like dessert is filled with nuts, sugar, and corn. The PUFAs content may be due to the use of PUFA-rich ingredients such as nuts. Therefore, *apam balik* could be considered a good source of PUFAs and may be beneficial to health, as frequent consumption of nuts has been demonstrated to have an association with lowering the risk of developing diet-related chronic diseases such as CVD [73,74]. However, the intake should also be controlled due to the sugar content. 

This study showed that deep-fried street foods tended to contain higher SFAs content than foods prepared using healthier methods, such as steaming, baking, stewing, grilling, pan-frying, and stir-frying. Deep-frying is the process of immersing food in hot oil between 130 °C and 190 °C [44]. Several studies compared the SFAs content of foods from different cooking methods. Choo et al. [75] found that deep-frying significantly increased the SFAs in a Japanese threadfin beam fillet (a type of fish) in comparison to grilling, baking, and steaming methods. Asmaa and Tajul [76] found that the SFAs content in chicken sausage significantly increased after deep-frying, whereas steaming in an oven did not affect the SFAs content in chicken sausage. These might explain the association between the deep-frying method and the SFAs content of street foods in this study. Given that Malaysia is one of the main producers of palm oil [77], and the high smoke point makes it suitable for deep-frying purposes, it is also worth noting that palm oil may account for the SFAs content in the studied deep-fried street foods. Notwithstanding little evidence that has managed to link the intake of saturated fats with health risks, deep-frying is a popular cooking method due to its fast preparation and ability to produce sensorily acceptable foods [78]. It is also the most preferred cooking method chosen by Malaysians [79]. In light of this, street food vendors in Malaysia should be advised to use as little oil as possible when preparing food. As suggested by the MDG 2020 [36], vendors could opt for using cooking methods that use less fat, such as grilling, pan-frying, and stir-frying instead of deep-frying, and they should also remove the excess oil after cooking. Besides that, they should also be aware not to reuse cooking oil more than twice to avoid imposing unwanted health effects on the consumers, as local food operators still have moderate awareness regarding the practice of reusing oil [39]. Given the limited amount of data currently available in the MyFCD on the FA composition in local, ready-to-eat dishes, the findings from this study could be used to update the database. Since the MyFCD is publicly available, the updated data could assist consumers in selecting street foods that are low in saturated fats and high in unsaturated fats.

## 5. Conclusions

This study demonstrated that SFAs content predominated in all three categories of street foods—main meals, snacks, and desserts, followed by MUFAs and PUFAs. Desserts contained the most SFAs, whereas snacks contained the most MUFAs and PUFAs. The majority of street foods that contained medium to high SFAs were deep-fried dishes, coconut milk-based dishes, and those that contained butter, processed meats, animal fats, or cheese. Meanwhile, street foods with low amounts of SFAs were prepared by steaming and stir-frying. Street foods that contained nuts had the highest content of MUFAs and PUFAs. Similarly, deep-fried street foods were also high in MUFAs. The local street foods contained no TFAs, possibly due to the limited use of partially hydrogenated fats such as ghee. The street foods that were prepared by deep-frying mostly contained high SFAs content, whereas, among the street foods that were prepared using healthier methods, most contained low SFAs content. Apart from updating the MyFCD database, these findings may encourage consumers to limit their consumption of coconut milk-based foods, deep-fried foods, and processed foods, as well as recognise several local street foods that may potentially increase their intake of unsaturated fats. Improving the nutritional composition of local street foods is imperative to creating a healthier food environment and reducing the development of diet-related chronic diseases. Educating street food vendors could be one of the ways to achieve this. Given that no amount of TFAs was detected in any of the street foods, especially those that are deep-fried, future studies could focus on the factors that may influence the TFAs content in foods, such as the frying temperature, duration of frying, usage of TFA-rich ingredients, and the practice of reusing oils. 

## Figures and Tables

**Table 1:** List of abbreviations and acronyms used in the paper.

Abbreviation	Definition
LMICs	Low- and middle-income countries
SFAs	Saturated fatty acids
MUFAs	Monounsaturated fatty acid
PUFAs	Polyunsaturated fatty acids
TFAs	Trans fatty acids
FAs	Fatty acids
LDL-C	Low-density lipoprotein-cholesterols
CVDs	Cardiovascular diseases
MyFCD	Malaysian Food Composition Database
FAO	Food and Agriculture Organization
GC-FID	Gas chromatography flame ionization detection
FAMEs	Fatty acid methyl esters
GC	Gas chromatograph
FID	Flame ionisation detector
LOD	Limit of detection
MDG	Malaysian Dietary Guidelines
SPSS	Statistical Package for Social Sciences

**Table 2:** Preparation method and fatty acid (FA) composition of 39 street foods according to high (>5 g/100 g food), medium (>1.5 g to ≤5 g/100 g food), and low (≤1.5 g/100 g food) SFAs ^1^ content classification [35].

High SFAs ^1^ Content (>5 g/100 g Food)							
No.	Name of Street Food (*n* = Number of States in which the Street Food Was Sampled)	Food Category	Preparation Method	Fatty Acid Composition (Mean ± SD)			
				SFAs ^1^		MUFAs ^2^	PUFAs ^3^
				g/100 g Food	Percentage (%)		
1	*Cakoi* (*n* = 2)	Snack	Deep-frying	24.3 ± 3.6	52.2 ± 7.8	37.6 ± 5.9	10.1 ± 1.7
2	Popcorn (*n* = 1)	Dessert	Grilling	19.0 ± 0.8	59.5 ± 1.3	27.5 ± 0.8	13.1 ± 0.5
3	*Kerepek* (*n* = 1)	Snack	Deep-frying	17.7 ± 0.1	41.1 ± 0.1	40.9 ± 0.2	18.0 ± 0.0
4	French fries (*n* = 1)	Snack	Deep-frying	8.3 ± 0.7	40.3 ± 1.1	37.3 ± 0.4	22.5 ± 0.6
5	Chicken nuggets (*n* = 5)	Snack	Deep-frying	8.0 ± 2.3	42.7 ± 1.8	40.4 ± 2.8	16.9 ± 4.1
6	Donut (*n* = 2)	Dessert	Deep-frying	7.4 ± 1.0	47.3 ± 1.8	40.0 ± 2.8	12.8 ± 1.1
7	*Kuih keria* (*n* = 1)	Dessert	Deep-frying	6.9 ± 0.2	45.0 ± 0.0	41.0 ± 0.0	14.0 ± 0.0
8	Fried *popiah* (*n* = 1)	Snack	Deep-frying	6.7 ± 0.0	46.9 ± 0.9	40.6 ± 0.2	12.6 ± 0.7
9	Curry puff (*n* = 5)	Snack	Deep-frying	6.7 ± 1.4	46.5 ± 1.8	41.0 ± 2.0	12.5 ± 1.8
10	Fried chicken with cheese (*n* = 1)	Snack	Deep-frying	6.3 ± 0.1	38.0 ± 0.0	41.0 ± 0.0	21.0 ± 0.0
11	Fried chicken (*n* = 3)	Snack	Deep-frying	6.2 ± 1.8	43.2 ± 3.3	42.2 ± 1.7	14.8 ± 3.7
12	Banana fritters with cheese (*n* = 1)	Dessert	Deep-frying	6.3 ± 0.7	45.3 ± 0.7	38.3 ± 0.4	16.4 ± 0.3
13	*Murtabak* (*n* = 1)	Snack	Pan-frying	5.4 ± 0.2	46.0 ± 0.0	43.0 ± 0.0	11.0 ± 0.0
14	Net crepes (*Roti jala*) (*n* = 1)	Main meal	Pan-frying	5.2 ± 0.1	53.4 ± 1.3	34.3 ± 1.9	12.4 ± 0.6
15	*Roti john* (*n* = 1)	Main meal	Pan-frying	5.1 ± 0.1	43.2 ± 0.3	37.9 ± 0.2	19.0 ± 0.5
Medium SFAs ^1^ content (>1.5 g to ≤5 g/100 g food)							
16	Pizza (*n* = 1)	Snack	Baking	4.5 ± 0.3	51.1 ± 0.7	15.6 ± 1.3	33.4 ± 2.1
17	*Nasi lemak* with fried chicken (*n* = 1)	Main meal	Steaming	4.3 ± 0.1	54.5 ± 0.7	32.5 ± 0.7	13.0 ± 0.0
18	Glutinous rice with *rendang* (meat cooked with spices) (*n* = 1)	Main meal	Steaming	4.3 ± 0.1	83.5 ± 0.7	11.7 ± 0.5	4.8 ± 1.2
19	Chicken burger (*n* = 1)	Main meal	Pan-frying	4.3 ± 0.1	44.0 ± 0.0	39.0 ± 0.0	17.0 ± 0.0
20	Takoyaki (*n* = 3)	Snack	Pan-frying	4.3 ± 0.7	33.6 ± 2.3	32.0 ± 2.9	34.5 ± 1.0
21	*Keropok lekor* (*n* = 2)	Snack	Deep-frying	4.1 ± 0.8	46.3 ± 1.1	38.3 ± 2.5	15.5 ± 3.5
22	Noodles with gravy (curry/soy sauce) (*n* = 2)	Main meal	Stewing	4.0 ± 0.0	50.0 ± 0.0	32.4 ± 0.2	17.7 ± 0.2
23	Banana fritters (*n* = 1)	Dessert	Deep-frying	4.0 ± 0.2	47.0 ± 0.0	41.0 ± 0.0	12.0 ± 0.0
24	Fried sausage (*n* = 3)	Snack	Deep-frying	3.7 ± 1.4	38.1 ± 2.5	37.3 ± 5.3	24.6 ± 7.3
25	*Roti canai* (flat bread) (*n* = 2)	Main meal	Pan-frying	3.6 ± 2.5	47.1 ± 1.3	37.8 ± 0.0	15.2 ± 0.3
26	Fried fish ball (*n* = 1)	Snack	Deep-frying	3.4 ± 0.1	42.7 ± 0.3	40.1 ± 0.2	12.7 ± 0.3
27	*Kuih seri muka* (*n* = 1)	Dessert	Steaming	3.1 ± 0.0	73.5 ± 0.7	18.5 ± 0.7	8.0 ± 0.0
28	*Apam balik* with cheese (*n* = 1)	Dessert	Pan-frying	3.0 ± 0.0	39.7 ± 2.2	21.9 ± 1.0	38.5 ± 1.2
29	Fried *kuey teow* (*n* = 1)	Main meal	Stir-frying	2.9 ± 0.3	45.0 ± 0.0	43.0 ± 0.0	12.0 ± 0.0
30	Kebab (*n* = 1)	Main meal	Grilling	2.6 ± 0.0	35.4 ± 1.7	32.7 ± 0.4	32.0 ± 1.3
31	*Nasi lemak* (*n* = 9)	Main meal	Steaming	2.3 ± 0.5	49.5 ± 7.3	34.7 ± 6.0	15.8 ± 3.3
32	Satay (*n* = 1)	Snack	Grilling	2.3 ± 0.1	37.0 ± 0.0	33.0 ± 0.0	30.0 ± 0.0
33	*Kuih jelurut* (*n* = 1)	Dessert	Steaming	2.2 ± 0.1	88.5 ± 0.7	7.0 ± 0.0	4.5 ± 0.7
34	Fried rice (*n* = 1)	Main meal	Stir-frying	2.1 ± 0.0	43.0 ± 0.0	43.0 ± 0.0	14.0 ± 0.0
35	Fried noodles (*n* = 2)	Main meal	Stir-frying	1.9 ± 0.8	42.8 ± 4.5	40.6 ± 0.9	16.5 ± 3.6
36	*Apam balik* (*n* = 3)	Dessert	Pan-frying	1.9 ± 0.6	29.3 ± 4.1	32.0 ± 5.9	38.8 ± 8.8
37	Fried chicken (nonmeat parts) (*n* = 1)	Snack	Deep-frying	1.7 ± 0.1	44.0 ± 0.0	25.5 ± 0.7	30.5 ± 0.7
Low SFAs ^1^ content (≤1.5 g/100 g food)							
38	Fried vermicelli @ fried *mihun* (*n* = 1)	Main meal	Stir-frying	1.4 ± 0.1	42.9 ± 0.6	44.4 ± 1.5	12.7 ± 0.9
39	Chicken rice (*n* = 2)	Main meal	Steaming	1.2 ± 0.3	43.6 ± 2.0	39.5 ± 0.7	16.9 ± 1.3

^1^ SFAs is the abbreviation for saturated fatty acids. ^2^ MUFAs is the abbreviation for monounsaturated fatty acids. ^3^ PUFAs is the abbreviation for polyunsaturated fatty acids.

**Table 3:** Association between preparation methods and SFAs ^1^ content of street foods (*n* = 70).

Category of Preparation Method (*n* = 70)	Category of SFAs ^1^ Content per 100 g of Food		Chi-Square Test		Symmetric Measures
	Low to Medium (5 g and Less)	High (More than 5 g)	χ^2^	*p*-Value	Φ
	*n* (%)				
Healthier preparation method ^2^ (*n* = 39)	34 (48.6)	5 (7.1)	24.65	<0.001 *	0.59
Less healthy preparation method (*n* = 31)	9 (12.9)	22 (31.4)			

* There is a significant difference (*p* < 0.05) based on the Chi-square test of association. ^1^ SFAs is the abbreviation for saturated fatty acids. ^2^ Steaming, baking, stewing, grilling, pan-frying, and stir-frying.

## Data Availability

Data is contained within the article or Supplementary Materials.

## Supplementary Materials

The following supporting information can be downloaded at: https://www.mdpi.com/article/10.3390/foods12061234/s1, Table S1: Fatty acid composition in 70 analysed street foods; Table S2: Explanation of each street food; Table S3: MUFA content in 39 street foods from highest to lowest amount; Table S4: PUFA content in 39 street foods from highest to lowest amount.

## References

[1] Ronto R., Wu J.H., Singh G.M. (2018). The Global Nutrition Transition: Trends, Disease Burdens and Policy Interventions. Public Health Nutr..

[2] Rajagopal (2010). Street Markets Influencing Urban Consumer Behavior in Mexico. Lat. Am. Bus. Rev..

[3] Khongtong J., Ab Karim M., Othman M., Bolong J. (2014). Consumption Pattern and Consumers’ Opinion toward Street Food in Nakhon Si Thammarat Province, Thailand. Int. Food Res. J..

[4] Chen J., Liu H. (2020). Nutritional Indices for Assessing Fatty Acids: A Mini-Review. Int. J. Mol. Sci..

[5] Albuquerque G., Lança De Morais I., Gelormini M., Sousa S., Casal S., Pinho O., Damasceno A., Moreira P., Breda J., Lunet N. (2022). Availability and Nutritional Composition of Street Food in Urban Central Asia: Findings from Almaty, Kazakhstan. Int. J. Public Health.

[6] Albuquerque G., Lança De Morais I., Gelormini M., Sousa S., Casal S., Pinho O., Moreira P., Breda J., Lunet N., Padrão P.J. (2020). Macronutrient Composition of Street Food in Central Asia: Bishkek, Kyrgyzstan. Food Sci. Nutr..

[7] Albuquerque G., Gelormini M., De Morais I.L., Sousa S., Casal S., Pinho O., Moreira P., Breda J., Lunet N., Padrão P. (2020). Street Food in Eastern Europe: A Perspective from an Urban Environment in Moldova. Br. J. Nutr..

[8] Albuquerque G., Morais I., Gelormini M., Casal S., Damasceno A., Pinho O., Moreira P., Jewell J., Breda J., Lunet N. (2019). Street Food in Dushanbe, Tajikistan: Availability and Nutritional Value. Br. J. Nutr..

[9] Sousa S., De Morais I.L., Albuquerque G., Gelormini M., Santos M., Filipović-Hadžiomeragić A., Stojisavljevic D., Damasceno A., Moreira P., Breda J. (2021). Nutritional Content of Street Food and Takeaway Food Purchased in Urban Bosnia and Herzegovina. Foods.

[10] Sousa S., Gelormini M., Damasceno A., Lopes S.A., Maló S., Chongole C., Muholove P., Casal S., Pinho O., Moreira P. (2019). Street Food in Maputo, Mozambique: Availability and Nutritional Value of Homemade Foods. Nutr. Health.

[11] Zaki H., Zaki M., Abdulla M., Abdel-Latif E.J. (2021). Evaluation of Fatty Acid Indices and Fatty Acid Content Including Trans Fat of Different Fried Food Types Using Gas-Liquid Chromatography Technique. Adv. Anim. Veter-Sci..

[12] Gupta V., Downs S.M., Ghosh-Jerath S., Lock K., Singh A. (2016). Unhealthy Fat in Street and Snack Foods in Low-Socioeconomic Settings in India: A Case Study of the Food Environments of Rural Villages and an Urban Slum. J. Nutr. Educ. Behav..

[13] Manzoor S., Masoodi F., Rashid R., Ahmad M., Ul Kousar M. (2022). Quality Assessment and Degradative Changes of Deep-Fried Oils in Street Fried Food Chain of Kashmir, India. Food Control.

[14] Bhat S., Maganja D., Huang L., Wu J.H., Marklund M.J.N. (2022). Influence of Heating During Cooking on Trans Fatty Acid Content of Edible Oils: A Systematic Review and Meta-Analysis. Nutrients.

[15] Manzoor S., Masoodi F., Rashid R. (2023). Influence of Food Type, Oil Type and Frying Frequency on the Formation of Trans-Fatty Acids During Repetitive Deep-Frying. Food Control.

[16] Cui Y., Hao P., Liu B., Meng X. (2017). Effect of Traditional Chinese Cooking Methods on Fatty Acid Profiles of Vegetable Oils. Food Chem..

[17] Chen Y., Yang Y., Nie S., Yang X., Wang Y., Yang M., Li C., Xie M. (2014). The Analysis of Trans Fatty Acid Profiles in Deep Frying Palm Oil and Chicken Fillets with an Improved Gas Chromatography Method. Food Control.

[18] Haron H., Zainal Arifen Z.N., Shahar S., Mohamad H., Mohd Yazid S.F.Z., Michael V., Abeyasinghe R., Taketo T., Trieu K.J.F. (2022). Street Food in Malaysia: What Are the Sodium Levels?. Foods.

[19] Mensink R.P., World Health Organization (2016). Effects of Saturated Fatty Acids on Serum Lipids and Lipoproteins: A Systematic Review and Regression Analysis.

[20] Ference B.A., Ginsberg H.N., Graham I., Ray K.K., Packard C.J., Bruckert E., Hegele R.A., Krauss R.M., Raal F.J., Schunkert H. (2017). Low-Density Lipoproteins Cause Atherosclerotic Cardiovascular Disease. 1. Evidence from Genetic, Epidemiologic, and Clinical Studies. A Consensus Statement from the European Atherosclerosis Society Consensus Panel. Eur. Heart J..

[21] Mensink R.P., Katan M.B. (1990). Effect of Dietary Trans Fatty Acids on High-Density and Low-Density Lipoprotein Cholesterol Levels in Healthy Subjects. N. Engl. J. Med..

[22] Schwingshackl L., Bogensberger B., Benčič A., Knüppel S., Boeing H., Hoffmann G. (2018). Effects of Oils and Solid Fats on Blood Lipids: A Systematic Review and Network Meta-Analysis. J. Lipid Res..

[23] Krauss R.M., Kris-Etherton P.M. (2020). Public Health Guidelines Should Recommend Reducing Saturated Fat Consumption as Much as Possible: Debate Consensus. Am. J. Clin. Nutr..

[24] Sacks F.M., Lichtenstein A.H., Wu J.H., Appel L.J., Creager M.A., Kris-Etherton P.M., Miller M., Rimm E.B., Rudel L.L., Robinson J.G. (2017). Dietary Fats and Cardiovascular Disease: A Presidential Advisory from the American Heart Association. Circulation.

[25] Murray C.J., Abbafati C., Abbas K.M., Abbasi M., Abbasi-Kangevari M., Abd-Allah F., Abdollahi M., Abedi P., Abedi A., Abolhassani H.J. (2020). Five Insights from the Global Burden of Disease Study 2019. Lancet.

[26] Abdullah N., Haron H., Abd Talib R., Nik W.N.N.W., Mohamed W. (2021). Proximate, Mineral and Fatty Acid Compositions of Healthy Recipes Used in Fit, Eat, Active, Training (Feat) Programme. Malays. J. Nutr..

[27] Akmar Z., Me N., Azimah R., Azlan A., Chan Y. (2013). The Trans Fatty Acids Content of Selected Foods in Malaysia. Malays. J. Nutr..

[28] Karupaiah T., Tan H.K., Ong W.W., Tan C.H., Sundram K.J. (2014). Trans Fatty Acid Content in Malaysian Supermarket Foods: A Field-to-Laboratory Approach in Assessing Food Risk. Food Addit. Contam. Part A.

[29] Malaysian Food Composition Database 1997 FCD Listing Fatty Acids in Ready-to-Eat Meals. https://myfcd.moh.gov.my/myfcd97/.

[30] Malaysian Food Composition Database History of Food Composition Database. https://myfcd.moh.gov.my/index.php/about-us/history-of-food-composition-database.html.

[31] FAO (2007). Promises and challenges of the informal food sector in developing countries. Rome: Food and Agriculture Organization. http://www.fao.org/3/a1124e/a1124e00.htm.

[32] International Scientific Committee (ISC) International Choices Criteria. https://www.choicesprogramme.org/our-work/nutrition-criteria/.

[33] Tee E.S., Ismail M.N., Nasir M.A., Khatijah I. (1997). Nutrient Composition of Malaysian Foods.

[34] Horwitz W. (2000). Official Methods of Analysis.

[35] Department of Health and the Food Standards Agency (2016). Guide to Creating a Front of Pack (FoP) Nutrition Label for Pre-Packed Products Sold through Retail Outlets. https://www.food.gov.uk/sites/default/files/media/document/fopguidance_0.pdf.

[36] National Coordinating Committee on Food and Nutrition (2021). Malaysian Dietary Guidelines 2020. Putrajaya: National Coordinating Committee on Food and Nutrition, Ministry of Health Malaysia. https://nutrition.moh.gov.my/MDG2020/mobile/index.html#p=1.

[37] Albuquerque G., Sousa S., Lança De Morais I., Gelormini M., Santos M., Moreira P., Damasceno A., Breda J., Lunet N., Padrão P. (2022). What Is the Sodium and Trans-Fat Content in Popular Street and Takeaway Food in Bosnia and Herzegovina?. J. Food Compos. Anal..

[38] Dorni C., Sharma P., Saikia G., Longvah T. (2018). Fatty Acid Profile of Edible Oils and Fats Consumed in India. Food Chem..

[39] Aziz A., Elias S., Sabran M.R. (2018). Repeatedly Heating Cooking Oil among Food Premise Operators in Bukit Mertajam, Pulau Pinang and Determination of Peroxide in Cooking Oil. Malays. J. Med. Health Sci..

[40] Malaysian Palm Oil Council Malaysian Palm Oil in the Professional Kitchen. https://mpoc.org.my/malaysian-palm-oil-in-the-professional-kitchen/.

[41] Parveez G.K.A., Hishamuddin E., Loh S.K., Ong-Abdullah M., Salleh K.M., Bidin M., Sundram S., Hasan Z.A.A., Idris Z.J.J. (2020). Oil Palm Economic Performance in Malaysia and R&D Progress in 2019. J. Oil Palm Res..

[42] Weber C., Harnack L., Johnson A., Jasthi B., Pettit J., Stevenson J.J. (2022). Nutrient Comparisons of Margarine/Margarine-like Products, Butter Blend Products and Butter in the Us Marketplace in 2020 Post-Fda Ban on Partially Hydrogenated Oils. Public Health Nutr..

[43] Liu Q., Rossouw J.E., Roberts M.B., Liu S., Johnson K.C., Shikany J.M., Manson J.E., Tinker L.F., Eaton C.B. (2017). Theoretical Effects of Substituting Butter with Margarine on Risk of Cardiovascular Disease. Epidemiology.

[44] Wroniak M., Raczyk M., Kruszewski B., Symoniuk E., Dach D.J. (2021). Effect of Deep Frying of Potatoes and Tofu on Thermo-Oxidative Changes of Cold Pressed Rapeseed Oil, Cold Pressed High Oleic Rapeseed Oil and Palm Olein. Antioxidants.

[45] Henry C.J., Kaur B., Quek R.Y.C. (2020). Are Asian Foods as “Fattening” as Western-Styled Fast Foods?. Eur. J. Clin. Nutr..

[46] Krauss R.M., Kris-Etherton P.M. (2020). Public Health Guidelines Should Recommend Reducing Saturated Fat Consumption as Much as Possible: No. Am. J. Clin. Nutr..

[47] Ismail S.R., Maarof S.K., Siedar Ali S., Ali A.J. (2018). Systematic Review of Palm Oil Consumption and the Risk of Cardiovascular Disease. PLoS ONE.

[48] Williams C.M., Salter A.J., Care M. (2016). Saturated Fatty Acids and Coronary Heart Disease Risk: The Debate Goes On. Curr. Opin. Clin. Nutr. Metab. Care.

[49] Wu J.H., Micha R., Mozaffarian D.J. (2019). Dietary Fats and Cardiometabolic Disease: Mechanisms and Effects on Risk Factors and Outcomes. Nat. Rev. Cardiol..

[50] Salleh R., Ganapathy S.S., Ibrahim Wong N., Cheong S.M., Ahmad M.H., Palaniveloo L., Othman F., Baharudin A., Megat Radzi M.R., Selamat R. (2021). Is Socio-Demographic Status, Body Mass Index, and Consumption of Food Away from Home Associated with High Sodium Intake among Adults in Malaysia?: Findings from the Malaysian Community Salt Survey (Mycoss). J. Health Popul. Nutr..

[51] Asiegbu C.V., Lebelo S.L., Tabit F.T. (2016). The Food Safety Knowledge and Microbial Hazards Awareness of Consumers of Ready-to-Eat Street-Vended Food. Food Control.

[52] Aluko O.O., Ojeremi T.T., Olaleke D.A., Ajidagba E.B. (2014). Evaluation of Food Safety and Sanitary Practices among Food Vendors at Car Parks in Ile Ife, Southwestern Nigeria. Food Control.

[53] Kelly M. (2016). The Nutrition Transition in Developing Asia: Dietary Change, Drivers and Health Impacts. Eating, Drinking: Surviving. The International Year of Global Understanding - IYGU.

[54] Hiamey S.E., Amuquandoh F.E., Boison G.A. (2013). Are We Indeed What We Eat? Street Food Consumption in the Market Circle Area of Takoradi, Ghana. Nutr. Health.

[55] Steyn N.P., Mchiza Z., Hill J., Davids Y.D., Venter I., Hinrichsen E., Opperman M., Rumbelow J., Jacobs P. (2014). Nutritional Contribution of Street Foods to the Diet of People in Developing Countries: A Systematic Review. Public Health Nutr..

[56] Hishamuddin E., Abd Razak R., Yeoh C., Ahmad Tarmizi A.J. (2022). Assessment of Trans Fatty Acid Levels in Refined Palm-Based Oils and Commercial Vegetable Oils in the Malaysian Market. J. Oil Palm Res..

[57] Mancini A., Imperlini E., Nigro E., Montagnese C., Daniele A., Orrù S., Buono P. (2015). Biological and Nutritional Properties of Palm Oil and Palmitic Acid: Effects on Health. Molecules.

[58] May C.Y., Nesaretnam K. (2014). Research Advancements in Palm Oil Nutrition. Eur. J. Lipid Sci. Technol..

[59] Alina A., Ah N.M., Shazamawati Z., Nurulhuda M., Hs U.S., Imtinan A.J. (2012). Effect of Grilling and Roasting on the Fatty Acids Profile of Chicken and Mutton. World Appl. Sci. J..

[60] Raji M.N.A., Ab Karim S., Ishak F.A.C., Arshad M.M. (2017). Past and Present Practices of the Malay Food Heritage and Culture in Malaysia. J. Ethn. Foods.

[61] Kamaruzaman M.Y.B., Ab Karim S., Ishak F.A.B.C., Arshad M.M.B. (2020). The Diversity of Traditional Malay Kuih in Malaysia and Its Potentials. J. Ethn. Foods.

[62] Lani M., Matsor N., Nasution Z., Ku P., Yusof A.J. (2015). Substitution Effects of Coconut Milk with Soymilk on Sensory Acceptance and Shelf Life Of’nasi Lemak’. Br. J. Appl. Sci. Technol..

[63] Bungsu S.H., Misdih M., Damit D., Yassin S.M. (2019). Identiti Budaya: Dalam Konteks Kuih Tradisional Etnik Brunei Di Sabah: Cultural Identity: In the Context of Sabah Brunei Ethnic Traditional Kuih. J. Borneo Soc. Transform. Stud..

[64] Mustafa M., Nagalingam S., Tye J., Shafii A.H., Dolah J. (2012). Looking Back to the Past: Revival of Traditional Food Packaging. 2nd Regional Conference on Local Knowledge (KEARIFAN TEMPATAN). Jerejak Isl..

[65] Marina A., Nurulazizah S.J. (2014). Use of Coconut Versus Dairy Milk Products in Malaysian Dishes: Comparison of Nutritional Composition and Sensory Evaluation. J. Food Nutr. Res..

[66] Górska-Warsewicz H., Rejman K., Laskowski W., Czeczotko M.J.N. (2019). Butter, Margarine, Vegetable Oils, and Olive Oil in the Average Polish Diet. Nutrients.

[67] Karupaiah T., Noor M.I., Sundram K. (2019). Dietary Fatty Acids and Their Influence on Blood Lipids and Lipoproteins. Healthful Lipids.

[68] Orsavova J., Misurcova L., Vavra Ambrozova J., Vicha R., Mlcek J.J. (2015). Fatty Acids Composition of Vegetable Oils and Its Contribution to Dietary Energy Intake and Dependence of Cardiovascular Mortality on Dietary Intake of Fatty Acids. Int. J. Mol. Sci..

[69] Bonku R., Yu J.J., Wellness H. (2020). Health Aspects of Peanuts as an Outcome of Its Chemical Composition. Food Sci. Hum. Wellness.

[70] Laskowski W., Górska-Warsewicz H., Kulykovets O.J. (2018). Meat, Meat Products and Seafood as Sources of Energy and Nutrients in the Average Polish Diet. Nutrients.

[71] Junaidah S., Fauzi F.A., Jamil J., Majid H.N.A. (2022). Dari Perlis ke Sabah: Warna-warni kuih-muih warisan Malaysia. Pelestarian Warisan Makanan Tradisional Melayu.

[72] Tarmizi S.F.M., Daud N.M., Rahman H.A. (2020). Malaysian Ready-to-Eat Cooked Dishes: Consumption Patterns among Adults and Nutrient Composition of Selected Highly Consumed Dishes. Malays. Appl. Biol..

[73] Aune D., Keum N., Giovannucci E., Fadnes L.T., Boffetta P., Greenwood D.C., Tonstad S., Vatten L.J., Riboli E., Norat T.J. (2016). Nut Consumption and Risk of Cardiovascular Disease, Total Cancer, All-Cause and Cause-Specific Mortality: A Systematic Review and Dose-Response Meta-Analysis of Prospective Studies. BMC Med..

[74] Zhou D., Yu H., He F., Reilly K.H., Zhang J., Li S., Zhang T., Wang B., Ding Y., Xi B.J. (2014). Nut Consumption in Relation to Cardiovascular Disease Risk and Type 2 Diabetes: A Systematic Review and Meta-Analysis of Prospective Studies. Am. J. Clin. Nutr..

[75] Choo P.Y., Azlan A., Khoo H.E. (2018). Cooking Methods Affect Total Fatty Acid Composition and Retention of Dha and Epa in Selected Fish Fillets. Sci. Asia.

[76] Asmaa A., Tajul A.Y. (2017). Influence of Superheated Steam and Deep Frying Cooking on the Proximate, Fatty Acids, and Amino Acids Composition of Chicken Sausage. Int. Food Res. J..

[77] Liberty J.T., Dehghannya J., Ngadi M.O. (2019). Effective Strategies for Reduction of Oil Content in Deep-Fat Fried Foods: A Review. Trends Food Sci. Technol..

[78] Bordin K., Tomihe Kunitake M., Kazue Aracava K. (2013). Silvia Favaro Trindade, C.J. Changes in Food Caused by Deep Fat Frying-a Review. Arch. Latinoam. Nutr..

[79] Ahmad N.I., Wan Mahiyuddin W.R., Tengku Mohamad T.R., Ling C.Y., Daud S.F., Hussein N.C., Abdullah N.A., Shaharudin R., Sulaiman L.H., Research N. (2016). Fish Consumption Pattern among Adults of Different Ethnics in Peninsular Malaysia. Food Nutr. Res..

