# Supplementary material for: Fatty Acid Composition of Selected Street Foods Commonly Available in Malaysia
Source: Foods. 2023 Mar 14;12(6):1234. doi: 10.3390/foods12061234 (PMC10048182; doi:10.3390/foods12061234)
Supplement: Supplementary file 1 [file foods-12-01234-s001.zip › Table S1_revised.pdf]

TABLE S1: FATTY ACID COMPOSITION IN 70 ANALYSED STREET FOODS

Supplementary table: Individual saturated fatty acid (SFA) content (% total fat) and fat content (g/100 g food) of 70 analysed street foods by state.

No.	Street food sample (*n* = 2) by state	Fat (g/100 g food)	SFA					
			Lauric	Myristic	Palmitic	Stearic	Myristoleic	Capric
			C 12:0	C 14:0	C 16:0	C 18:0	C 14:1	C 10:0
**SELANGOR**								
1	*Nasi lemak*	7.8 ± 0.1	6.955 ± 0.375	3.210 ± 0.085	28.300 ± 0.283	4.075 ± 4.335	<0.100	0.942 ± 0.059
2	Pizza	8.9 ± 0.5	3.445 ± 0.078	6.005 ± 0.092	28.000 ± 0.000	7.795 ± 0.092	0.563 ± 0.013	2.050 ± 0.071
3	Chicken nuggets	19.4 ± 0.1	0.348 ± 0.030	0.948 ± 0.045	34.400 ± 0.424	4.580 ± 0.042	<0.100	<0.100
4	Curry puff	15.8 ± 0.4	1.215 ± 0.021	1.300 ± 0.000	39.000 ± 0.141	3.825 ± 0.021	<0.100	0.108 ± 0.002
5	*Apam balik*	7.5 ± 0.6	1.545 ± 0.007	0.798 ± 0.011	20.650 ± 0.071	3.570 ± 0.042	<0.100	0.131 ± 0.016
**KUALA LUMPUR**								
1	*Nasi lemak*	4.7 ± 0.1	12.850 ± 0.778	5.805 ± 0.191	26.300 ± 0.424	4.505 ± 0.064	<0.100	1.665 ± 0.205
2	Kebab	7.4 ± 0.3	1.260 ± 0.311	1.165 ± 0.120	26.550 ± 0.495	6.295 ± 0.035	<0.100	<0.100
3	Takoyaki	12.7 ± 0.1	0.198 ± 0.002	0.814 ± 0.028	27.800 ± 0.283	7.060 ± 0.042	<0.100	<0.100
4	Banana fritters with cheese	13.9 ± 1.3	0.993 ± 0.039	1.805 ± 0.007	38.050 ± 0.354	4.025 ± 0.007	<0.100	0.264 ± 0.005
5	*Apam balik*	4.6 ± 0.8	<0.100	<0.100	21.000 ± 0.283	3.825 ± 0.049	<0.100	<0.100
**NEGERI SEMBILAN**								
1	*Roti john*	11.9 ± 0.1	0.766 ± 0.063	0.968 ± 0.045	34.150 ± 0.354	6.645 ± 0.092	0.036 ± 0.008	0.072 ± 0.004
2	Noodles with gravy (curry / soy sauce)	4.0 ± 0.0	12.600 ± 0.141	5.245 ± 0.007	24.650 ± 0.071	4.660 ± 0.014	<0.100	1.670 ± 0.042
3	*Nasi lemak*	4.2 ± 0.4	10.600 ± 0.141	4.695 ± 0.007	25.100 ± 0.000	4.990 ± 0.014	<0.100	1.365 ± 0.035
4	Fried sausage	9.0 ± 0.3	0.223 ± 0.009	0.860 ± 0.013	29.450 ± 0.354	5.980 ± 0.071	0.099 ± 0.009	<0.100
5	*Apam balik* with cheese	7.6 ± 0.4	1.930 ± 0.141	4.350 ± 0.156	24.850 ± 0.212	6.075 ± 0.021	<0.100	1.285 ± 0.064
**MELAKA**								
1	*Nasi lemak*	3.9 ± 0.1	7.830 ± 0.693	3.655 ± 0.191	27.850 ± 0.212	4.335 ± 0.120	<0.100	1.045 ± 0.120
2	Chicken nuggets	13.6 ± 0.0	0.501 ± 0.063	0.981 ± 0.042	35.050 ± 0.071	5.245 ± 0.035	<0.100	<0.100
3	French fries	20.5 ± 1.3	0.411 ± 0.049	0.901 ± 0.006	35.000 ± 0.849	4.350 ± 0.042	<0.100	<0.100
4	Fried sausage	6.8 ± 0.0	<0.100	1.275 ± 0.106	24.650 ± 0.071	10.550 ± 0.071	<0.100	<0.100
5	*Apam balik*	6.8 ± 0.4	4.070 ± 0.127	1.725 ± 0.049	22.700 ± 0.141	3.495 ± 0.035	<0.100	0.373 ± 0.025
**PERAK**								
1	*Roti canai* (flat bread)	3.9 ± 0.0	<0.100	<0.100	2.350 ± 1.202	0.250 ± 0.071	<0.100	<0.100
2	Net crepes (*Roti jala*)	9.8 ± 0.5	0.850 ± 0.071	0.350 ± 0.071	3.300 ± 0.141	0.350 ± 0.071	<0.100	<0.100

3	Fried *popiah*	14.3 ± 0.3	<0.100	0.200 ± 0.000	5.900 ± 0.000	0.500 ± 0.000	<0.100	<0.100
4	*Kerepek*	43.1 ± 0.5	0.500 ± 0.000	0.500 ± 0.000	14.400 ± 0.141	1.550 ± 0.071	<0.100	<0.100
5	Popcorn	31.9 ± 0.6	6.000 ± 0.0424	1.650 ± 0.071	8.450 ± 0.071	1.100 ± 0.000	<0.100	0.650 ± 0.071
**PENANG**								
1	*Nasi lemak*	4.4 ± 0.4	0.100 ± 0.000	<0.100	1.400 ± 0.141	0.200 ± 0.000	<0.100	<0.100
2	Noodles with gravy (curry / soy sauce)	4.0 ± 0.0	0.500 ± 0.000	0.200 ± 0.000	1.100 ± 0.000	0.200 ± 0.000	<0.100	<0.100
3	Fried noodles	3.2 ± 0.2	<0.100	<0.100	1.150 ± 0.071	0.100 ± 0.000	<0.100	<0.100
4	*Cakoi*	25.4 ± 0.6	7.800 ± 0.141	3.250 ± 0.071	<0.100	1.200 ± 0.000	<0.100	1.000 ± 0.000
5	Curry puff	15.9 ± 0.2	0.100 ± 0.000	0.200 ± 0.000	6.300 ± 0.141	0.700 ± 0.000	<0.100	<0.100
**KEDAH**								
1	*Nasi lemak*	4.0 ± 0.2	2.705 ± 0.049	1.890 ± 0.028	35.500 ± 0.707	5.000 ± 0.000	<0.100	0.375 ± 0.007
2	Chicken nuggets	23.0 ± 0.1	0.210 ± 0.014	0.840 ± 0.028	36.500 ± 0.707	4.600 ± 0.000	<0.100	<0.100
3	Fried sausage	12.6 ± 0.1	0.205 ± 0.007	0.900 ± 0.000	34.000 ± 0.000	5.000 ± 0.000	<0.100	<0.100
4	Curry puff	17.3 ± 0.1	0.900 ± 0.000	1.095 ± 0.134	41.000 ± 0.000	4.060 ± 0.085	<0.100	<0.100
5	Donut	17.6 ± 0.2	0.285 ± 0.007	0.900 ± 0.000	40.000 ± 0.000	4.000 ± 0.000	<0.100	<0.100
**PERLIS**								
1	Chicken rice	2.3 ± 0.1	<0.100	<0.100	0.800 ± 0.000	0.100 ± 0.000	<0.100	<0.100
2	Fried chicken	14.4 ± 0.4	<0.100	0.100 ± 0.000	5.000 ± 0.000	0.725 ± 0.035	<0.100	<0.100
3	Chicken nuggets	24.7 ± 0.8	<0.100	0.200 ± 0.000	9.300 ± 0.283	1.050 ± 0.071	<0.100	<0.100
4	Takoyaki	10.8 ± 0.3	<0.100	<0.100	2.850 ± 0.071	0.500 ± 0.000	<0.100	<0.100
5	Fried fish ball	7.9 ± 0.2	<0.100	0.100 ± 0.000	2.850 ± 0.071	0.400 ± 0.000	<0.100	<0.100
**KELANTAN**								
1	Fried vermicelli @ fried *mihun*	3.2 ± 0.2	<0.100	<0.100	1.150 ± 0.071	0.150 ± 0.071	<0.100	<0.100
2	*Nasi lemak*	4.7 ± 0.2	0.450 ± 0.071	0.200 ± 0.000	1.250 ± 0.071	0.200 ± 0.000	<0.100	<0.100
3	Glutinous rice with *rendang* (meat cooked with spices)	5.2 ± 0.2	1.850 ± 0.071	0.850 ± 0.071	0.800 ± 0.000	0.200 ± 0.000	<0.100	0.200 ± 0.000
4	*Cakoi*	20.5 ± 0.8	0.100 ± 0.000	0.200 ± 0.000	8.200 ± 0.283	0.850 ± 0.071	<0.100	<0.100
5	Curry puff	11.1 ± 0.1	<0.100	<0.100	4.350 ± 0.071	0.500 ± 0.000	<0.100	<0.100
**TERENGGANU**								
1	*Nasi lemak*	3.3 ± 0.1	20.000 ± 0.000	9.000 ± 0.000	20.000 ± 0.000	4.000 ± 0.000	<0.100	3.000 ± 0.000
2	Fried noodles	5.4 ± 0.1	0.265 ± 0.007	1.075 ± 0.007	40.000 ± 0.000	4.000 ± 0.000	<0.100	<0.100
3	Fried chicken	11.1 ± 0.2	0.260 ± 0.028	1.020 ± 0.028	34.500 ± 0.707	4.750 ± 0.354	0.140 ± 0.014	<0.100
4	Chicken nuggets	12.4 ± 0.1	0.280 ± 0.000	1.000 ± 0.000	38.000 ± 0.000	4.000 ± 0.000	<0.100	<0.100
5	Donut	13.8 ± 0.0	2.000 ± 0.000	2.000 ± 0.000	41.000 ± 0.000	4.000 ± 0.000	<0.100	0.145 ± 0.007
**PAHANG**								

1	Chicken rice	3.2 ± 0.0	1.660 ± 0.014	0.815 ± 0.926	35.500 ± 0.707	5.000 ± 0.000	<0.100	0.160 ± 0.000
2	*Nasi lemak*	4.6 ± 0.0	6.500 ± 0.707	3.000 ± 0.000	30.000 ± 0.000	5.000 ± 0.000	<0.100	0.855 ± 0.021
3	Fried chicken	17.5 ± 0.5	0.195 ± 0.007	0.985 ± 0.021	41.000 ± 0.000	4.150 ± 0.212	<0.100	<0.100
4	Curry puff	11.6 ± 0.2	0.745 ± 0.035	1.000 ± 0.000	41.000 ± 0.000	4.000 ± 0.000	<0.100	<0.100
5	Takoyaki	14.7 ± 0.0	0.190 ± 0.000	0.625 ± 0.035	25.500 ± 0.0707	5.100 ± 0.141	<0.100	<0.100
**JOHOR**								
1	*Nasi lemak* with fried chicken	7.9 ± 0.0	13.000 ± 0.000	6.000 ± 0.000	27.000 ± 0.000	4.000 ± 0.000	<0.100	2.000 ± 0.000
2	Fried *kuey teow*	6.5 ± 0.6	0.165 ± 0.007	0.880 ± 0.000	38.000 ± 0.000	4.000 ± 0.000	<0.100	<0.100
3	Fried chicken with cheese	17.2 ± 0.4	0.440 ± 0.000	0.900 ± 0.000	31.000 ± 0.000	5.000 ± 0.000	<0.100	<0.100
4	*Keropok lekor*	7.4 ± 0.1	0.405 ± 0.007	2.000 ± 0.000	20.000 ± 26.870	5.000 ± 0.000	<0.100	<0.100
5	*Kuih seri muka*	4.2 ± 0.1	30.500 ± 0.707	10.000 ± 0.000	20.000 ± 0.000	4.000 ± 0.000	<0.100	4.000 ± 0.000
**SABAH**								
1	*Murtabak*	11.7 ± 0.4	0.205 ± 0.007	0.900 ± 0.000	40.000 ± 0.000	5.000 ± 0.000	<0.100	<0.100
2	*Keropok lekor*	10.3 ± 0.6	0.460 ± 0.000	2.000 ± 0.000	38.000 ± 0.000	5.000 ± 0.000	<0.100	<0.100
3	Fried chicken (non-meat parts)	3.9 ± 0.3	0.340 ± 0.014	0.610 ± 0.000	27.000 ± 0.000	10.000 ± 0.000	<0.100	<0.100
4	Satay	6.1 ± 0.1	0.195 ± 0.021	0.650 ± 0.000	24.000 ± 0.000	10.000 ± 0.000	0.115 ± 0.007	<0.100
5	*Kuih jelurut*	2.5 ± 0.1	40.000 ± 0.000	20.000 ± 0.000	10.000 ± 0.000	3.285 ± 0.000	<0.100	6.000 ± 0.000
**SARAWAK**								
1	Chicken burger	9.8 ± 0.3	0.160 ± 0.000	1.015 ± 0.021	35.000 ± 0.000	8.000 ± 0.000	0.155 ± 0.007	<0.100
2	*Roti canai* (flat bread)	11.0 ± 0.4	1.000 ± 0.000	1.000 ± 0.000	41.000 ± 0.000	4.000 ± 0.000	<0.100	<0.100
3	Fried rice	4.9 ± 0.1	0.165 ± 0.007	0.865 ± 0.007	36.000 ± 0.000	5.000 ± 0.000	<0.100	<0.100
4	Banana fritters	8.5 ± 0.4	2.000 ± 0.000	2.000 ± 0.000	38.000 ± 0.000	4.000 ± 0.000	<0.100	0.195 ± 0.007
5	*Kuih keria*	15.3 ± 0.4	0.330 ± 0.000	1.000 ± 0.000	40.000 ± 0.000	4.000 ± 0.000	<0.100	<0.100

Data represent average of two independent readings ± standard deviation; SFA – Saturated fatty acid.

Supplementary table: Individual monounsaturated fatty acid (MUFA) and polyunsaturated fatty acid (PUFA) contents (% total fat) of 70 analysed street foods by state.

No.	Street food sample (n = 2) by state	MUFA		PUFA			
		Palmitoleic	Oleic	Linoleic	Linolenic	Arachidic	Arachidonic
		C 16:1	C 18:1	C 18:2	C 18:3	C 20:0	C 20:4
**SELANGOR**							
1	*Nasi lemak*	1.285 ± 0.007	29.300 ± 0.141	16.400 ± 0.000	0.645 ± 0.021	0.190 ± 0.010	2.495 ± 0.106
2	Pizza	0.918 ± 0.023	14.950 ± 0.071	28.950 ± 0.071	1.905 ± 0.078	<0.100	<0.100
3	Chicken nuggets	1.515 ± 0.049	37.200 ± 0.141	18.550 ± 0.071	<0.100	0.210 ± 0.003	0.725 ± 0.012
4	Curry puff	0.279 ± 0.010	38.050 ± 0.071	14.950 ± 0.071	0.581 ± 0.012	0.279 ± 0.006	<0.100
5	*Apam balik*	0.156 ± 0.006	34.250 ± 0.071	34.850 ± 0.071	0.674 ± 0.037	0.840 ± 0.034	<0.100
**KUALA LUMPUR**							
1	*Nasi lemak*	0.632 ± 0.097	32.200 ± 0.283	13.050 ± 0.354	<0.100	<0.100	<0.100
2	Kebab	2.020 ± 0.141	30.750 ± 0.778	28.800 ± 0.566	2.335 ± 0.205	<0.100	0.792 ± 0.047
3	Takoyaki	0.744 ± 0.019	27.600 ± 0.000	26.750 ± 0.071	2.135 ± 0.021	0.295 ± 0.021	1.820 ± 0.071
4	Banana fritters with cheese	0.283 ± 0.018	37.500 ± 0.283	14.550 ± 0.071	1.250 ± 0.014	0.289 ± 0.007	<0.100
5	*Apam balik*	<0.100	25.200 ± 0.000	46.950 ± 0.354	1.840 ± 0.085	<0.100	<0.100
**NEGERI SEMBILAN**							
1	*Roti john*	1.215 ± 0.035	35.100 ± 0.283	17.300 ± 0.141	0.808 ± 0.013	0.216 ± 0.011	1.235 ± 0.035
2	Noodles with gravy (curry / soy sauce)	0.780 ± 0.005	29.350 ± 0.212	16.400 ± 0.000	1.145 ± 0.021	0.193 ± 0.008	0.625 ± 0.018
3	*Nasi lemak*	1.105 ± 0.007	31.400 ± 0.000	16.100 ± 0.000	0.395 ± 0.002	0.398 ± 0.017	0.613 ± 0.008
4	Fried sausage	2.660 ± 0.028	35.100 ± 0.141	21.400 ± 0.000	1.290 ± 0.028	0.179 ± 0.013	1.415 ± 0.007
5	*Apam balik* with cheese	<0.100	20.750 ± 0.071	35.850 ± 0.071	1.790 ± 0.028	<0.100	<0.100
**MELAKA**							
1	*Nasi lemak*	0.607 ± 0.042	34.200 ± 0.707	16.700 ± 0.283	0.460 ± 0.011	0.501 ± 0.015	0.180 ± 0.011
2	Chicken nuggets	0.923 ± 0.036	35.050 ± 0.212	19.800 ± 0.000	0.971 ± 0.070	<0.100	1.335 ± 0.148
3	French fries	<0.100	37.100 ± 0.283	19.700 ± 0.283	2.555 ± 0.290	<0.100	<0.100
4	Fried sausage	3.370 ± 0.028	27.400 ± 0.000	22.950 ± 0.071	1.495 ± 0.049	<0.100	5.940 ± 0.071
5	*Apam balik*	0.160 ± 0.039	33.050 ± 0.212	30.800 ± 0.141	0.665 ± 0.242	<0.100	<0.100
**PERAK**							
1	*Roti canai* (flat bread)	<0.100	1.400 ± 0.000	0.600 ± 0.000	<0.100	<0.100	<0.100
2	Net crepes (*Roti jala*)	<0.100	3.300 ± 0.283	1.150 ± 0.071	<0.100	<0.100	<0.100

3	Fried *popiah*	<0.100	5.700 ± 0.141	1.750 ± 0.071	<0.100	<0.100	<0.100
4	*Kerepek*	<0.100	17.250 ± 0.212	7.650 ± 0.071	<0.100	0.200 ± 0.000	<0.100
5	Popcorn	<0.100	8.750 ± 0.071	3.850 ± 0.071	0.200 ± 0.000	<0.100	<0.100
**PENANG**							
1	*Nasi lemak*	<0.100	1.700 ± 0.141	0.550 ± 0.071	<0.100	<0.100	<0.100
2	Noodles with gravy (curry / soy sauce)	<0.100	1.200 ± 0.000	0.600 ± 0.000	<0.100	<0.100	<0.100
3	Fried noodles	<0.100	1.250 ± 0.071	0.600 ± 0.000	<0.100	<0.100	<0.100
4	*Cakoi*	<0.100	8.400 ± 0.283	2.150 ± 0.071	<0.100	<0.100	<0.100
5	Curry puff	<0.100	6.550 ± 0.071	1.800 ± 0.000	<0.100	<0.100	<0.100
**KEDAH**							
1	*Nasi lemak*	0.860 ± 0.000	39.000 ± 0.000	10.000 ± 0.000	0.370 ± 0.000	0.340 ± 0.014	0.325 ± 0.021
2	Chicken nuggets	1.200 ± 0.000	41.500 ± 0.707	14.000 ± 0.000	0.585 ± 0.021	0.325 ± 0.007	<0.100
3	Fried sausage	1.000 ± 0.000	40.000 ± 0.000	16.000 ± 0.000	0.680 ± 0.014	0.285 ± 0.007	0.240 ± 0.000
4	Curry puff	0.200 ± 0.000	41.000 ± 0.000	10.500 ± 0.707	0.245 ± 0.035	0.360 ± 0.000	<0.100
5	Donut	0.200 ± 0.000	42.000 ± 0.000	12.000 ± 0.000	<0.100	0.355 ± 0.007	<0.100
**PERLIS**							
1	Chicken rice	<0.100	0.850 ± 0.071	0.300 ± 0.000	<0.100	<0.100	<0.100
2	Fried chicken	0.200 ± 0.000	5.550 ± 0.212	2.300 ± 0.141	0.100 ± 0.000	<0.100	0.100 ± 0.000
3	Chicken nuggets	0.100 ± 0.000	10.350 ± 0.354	3.200 ± 0.141	<0.100	<0.100	<0.100
4	Takoyaki	<0.100	3.550 ± 0.071	3.200 ± 0.141	0.300 ± 0.000	<0.100	<0.100
5	Fried fish ball	<0.100	3.150 ± 0.071	0.900 ± 0.000	<0.100	<0.100	<0.100
**KELANTAN**							
1	Fried vermicelli @ fried *mihun*	<0.100	1.350 ± 0.071	0.400 ± 0.000	<0.100	<0.100	<0.100
2	*Nasi lemak*	<0.100	1.600 ± 0.000	0.600 ± 0.000	<0.100	<0.100	<0.100
3	Glutinous rice with *rendang* (meat cooked with spices)	<0.100	0.600 ± 0.000	0.200 ± 0.000	<0.100	<0.100	<0.100
4	*Cakoi*	<0.100	8.450 ± 0.354	2.250 ± 0.071	<0.100	<0.100	<0.100
5	Curry puff	<0.100	4.650 ± 0.071	1.300 ± 0.000	<0.100	<0.100	<0.100
**TERENGGANU**							
1	*Nasi lemak*	0.560 ± 0.014	20.000 ± 0.000	8.000 ± 0.000	0.305 ± 0.021	0.295 ± 0.007	0.255 ± 0.007
2	Fried noodles	0.405 ± 0.021	40.000 ± 0.000	10.000± 0.000	0.465 ± 0.007	0.345 ± 0.007	<0.100
3	Fried chicken	3.050 ± 0.071	40.500 ± 0.707	13.000 ± 0.000	0.495 ± 0.007	0.235 ± 0.007	0.270 ± 0.168
4	Chicken nuggets	1.000 ± 0.000	40.000 ± 0.000	13.000 ± 0.000	0.435 ± 0.007	0.275 ± 0.007	0.185 ± 0.007
5	Donut	0.295 ± 0.007	37.000 ± 0.000	13.000 ± 0.000	0.315 ± 0.092	0.250 ± 0.000	<0.100
**PAHANG**							

1	Chicken rice	1.515 ± 0.021	38.000 ± 0.000	10.000 ± 0.000	0.735 ± 0.021	0.280 ± 0.028	0.320 ± 0.000
2	*Nasi lemak*	0.700 ± 0.000	35.000 ± 0.000	10.000 ± 0.000	0.455 ± 0.007	0.385 ± 0.007	0.270 ± 0.000
3	Fried chicken	0.405 ± 0.007	41.000 ± 0.000	11.000 ± 0.000	0.235 ± 0.007	0.335 ± 0.007	0.225 ± 0.007
4	Curry puff	0.250 ± 0.000	40.000 ± 0.000	12.000 ± 0.000	0.310 ± 0.000	0.335 ± 0.007	<0.100
5	Takoyaki	0.520 ± 0.014	32.000 ± 0.000	31.000 ± 0.000	3.150 ± 0.212	0.335 ± 0.007	0.165 ± 0.007
**JOHOR**							
1	*Nasi lemak* with fried chicken	0.855 ± 0.007	31.500 ± 0.707	10.000 ± 0.000	0.320 ± 0.000	0.360 ± 0.000	0.210 ± 0.000
2	Fried *kuey teow*	0.530 ± 0.000	42.000 ± 0.000	10.000 ± 0.000	0.355 ± 0.007	0.360 ± 0.000	0.135 ± 0.007
3	Fried chicken with cheese	2.000 ± 0.000	39.000 ± 0.000	18.000 ± 0.000	1.000 ± 0.000	0.315 ± 0.021	0.330 ± 0.000
4	*Keropok lekor*	1.000 ± 0.000	38.000 ± 0.000	9.000 ± 0.000	0.215 ± 0.007	0.420 ± 0.000	0.385 ± 0.007
5	*Kuih seri muka*	0.475 ± 0.021	20.000 ± 0.000	7.000 ± 0.000	0.260 ± 0.000	0.170 ± 0.000	0.265 ± 0.007
**SABAH**							
1	*Murtabak*	0.315 ± 0.007	42.000 ± 0.000	11.000 ± 0.000	0.345 ± 0.035	0.400 ± 0.028	<0.100
2	*Keropok lekor*	0.620 ± 0.014	36.000 ± 0.000	10.000 ± 0.000	0.395 ± 0.007	0.315 ± 0.007	0.600 ± 0.014
3	Fried chicken (non-meat parts)	1.035 ± 0.007	21.500 ± 2.121	20.000 ± 0.000	0.445 ± 0.021	0.275 ± 0.007	10.000 ± 0.000
4	Satay	3.000 ± 0.000	30.000 ± 0.000	20.000 ± 0.000	1.050 ± 0.042	0.215 ± 0.035	6.000 ± 0.000
5	*Kuih jelurut*	<0.100	6.500 ± 0.707	4.315 ± 0.092	0.185 ± 0.007	<0.100	<0.100
**SARAWAK**							
1	Chicken burger	2.000 ± 0.000	36.000 ± 0.000	15.000 ± 0.000	0.850 ± 0.014	0.215 ± 0.007	0.930 ± 0.000
2	*Roti canai* (flat bread)	0.195 ± 0.007	36.000 ± 0.000	14.000 ± 0.000	0.440 ± 0.028	0.310 ± 0.000	<0.100
3	Fried rice	6.825 ± 7.884	41.000 ± 0.000	10.000 ± 0.000	0.370 ± 0.028	0.325 ± 0.007	0.265 ± 0.007
4	Banana fritters	0.215 ± 0.007	40.000 ± 0.000	11.000 ± 1.414	0.670 ± 0.000	0.350 ± 0.000	<0.100
5	*Kuih keria*	0.190 ± 0.000	40.000 ± 0.000	13.000 ± 0.000	0.450 ± 0.014	0.320 ± 0.000	<0.100

Data represent average of two independent readings ± standard deviation; MUFA – Monounsaturated fatty acid; PUFA – Polyunsaturated fatty acid.
