# Supplementary material for: Fatty Acid Composition of Selected Street Foods Commonly Available in Malaysia
Source: Foods. 2023 Mar 14;12(6):1234. doi: 10.3390/foods12061234 (PMC10048182; doi:10.3390/foods12061234)
Supplement: Supplementary file 1 [file foods-12-01234-s001.zip › Table S2_revised.pdf]

TABLE S2: EXPLANATION OF EACH STREET FOOD

Supplementary table: Description of 14 selected street foods under the main meals category in Malaysia.

No.	Name of street food	Description of street food
1	Fried noodles	Yellow noodles that are stir-fried in sauces such as soy sauce, thick soy sauce, oyster sauce, and chili sauce. This dish is sometimes eaten with pickled chili.
2	Kebab	A pita bread with grilled chicken fillings, and topped with chili sauce, mayonnaise and seasoning sauce.
3	Chicken burger	Malaysian burger comprised of a ready-made chicken burger patty that is grilled with butter, and placed between two buns that are buttered and grilled. The patty is then topped with sauces such as chili sauce, mayonnaise, and seasoning sauce.
4	Fried *kuey teow*	Flat rice noodles that is stir-fried in sauces such as soy sauce, thick soy sauce, and oyster sauce. This dish is sometimes eaten with pickled chili.
5	*Roti john*	An omelette sandwich comprised of an oblong-shaped bread that is sliced halfway in the middle, cooked on an omelette, and topped with sauces such as chili sauce, mayonnaise, and seasoning sauce.
6	Fried vermicelli @ fried *mihun*	Rice vermicelli that are stir-fried in sauces such as soy sauce, thick soy sauce, oyster sauce, and chili sauce. This dish is sometimes eaten with chili sauce.
7	Fried rice	Overnight rice that is stir-fried with sauces such as soy sauce and oyster sauce. This dish is sometimes eaten with a soy sauce sambal.
8	*Nasi lemak* with fried chicken	A Malay dish comprised of fragrant rice cooked in a coconut milk and pandan leaves. Rice is served with a sambal (a mixture of chili paste and shrimp paste, garlic, ginger, shallot), toasted peanuts, deep-fried anchovies, cucumber and deep-fried chicken marinated with spices.
9	Noodles with gravy (Curry / Soy sauce)	Noodles served in coconut-based curry gravy, or tossed with a sauce mixture (thick soy sauce and oyster sauce).
10	Chicken rice	A dish comprised of yellow-coloured seasoned rice that is served with roasted or poached chicken, cucumber, its own chili sambal, light soy sauce and a clear soup.
11	*Nasi lemak*	A Malay dish comprised of fragrant rice cooked in a coconut milk and pandan leaves. Rice is served with a sambal (a mixture of chili paste and shrimp paste, garlic, ginger, shallot), toasted peanuts, deep-fried anchovies, cucumber and hard-boiled egg.
12	*Roti canai* (flat bread)	An Indian flatbread dish that is often served with *dhal* (lentil

		curry), fish curry, and sambal.
13	Glutinous rice with *rendang* (meat cooked with spices)	A Malay dish comprised of glutinous rice that is steamed with coconut milk, salt, and sugar, and served with either chicken or beef cooked with spices.
14	Net crepes *(Roti jala)*	A Malay dish made of a thin plain batter that is pan-fried without oil. The batter is poured into a funnel and onto a pan, to create a 'net.' This dish is eaten with meat curry.

Supplementary table: Description of 16 selected street foods under the snacks category in Malaysia.

No.	Name of street food	Description of street food
1	Fried fish ball	A mixture of processed fish meat with salt, sugar, monosodium glutamate (MSG), and chicken seasoning, made into balls and then deep-fried.
2	*Keropok lekor*	Signature snack from Terengganu that is made of a mixture of sea fish flesh with sago flour, salt, sugar, MSG, and ice-cold water. The mixture is shaped into a sausage and deep-fried. This snack can be eaten on its own or with a chili sauce.
3	Fried chicken with cheese	Processed chicken that is then added with cheese and deep-fried.
4	Fried sausage	Processed chicken shaped into sausage and then deep-fried.
5	Chicken nuggets	A mixture of processed chicken meat with salt, sugar, MSG, and coated with breadcrumbs. The nuggets are the commonly shaped into hearts and then deep-fried.
6	*Kerepek*	Deep-fried savoury crackers made from rice flour.
7	Takoyaki	A Japanese snack made of a wheat flour-based batter and cooked in a sphere molded pan. This snack is typically filled with minced or diced octopus, and green onion. In Malaysia, this snack is garnished with bonito flakes and drenched with takoyaki sauce and mayonnaise.
8	Pizza	An Italian dish made of a flat, round wheat-based dough and is commonly topped with tomato sauced, cheese, and various other toppings. The dough is then baked in a high-temperature oven along with the toppings.
9	Fried chicken	Chicken pieces that are coated in a seasoned batter and then deep-fried.
10	Fried chicken (non-meat parts)	Chicken pieces such as gizzards that are coated in a seasoned batter and then deep-fried.

11	Satay	Meat that is seasoned and skewered, and then grilled. This dish is often eaten with a savoury and spicy peanut sauce, onion, and cucumber.
12	*Cakoi*	A plain Chinese snack made of wheat-based dough that is cooked by deep-frying.
13	Fried *popiah*	Deep-fried spring rolls with vegetables fillings. This snack can be eaten on its own or with a sambal. The sambal sometimes contain dried shrimp.
14	Curry puff	A deep-fried snack made of a small pie with potato curry fillings.
15	*Murtabak*	A square-shaped, pan-fried pancake that is stuffed with minced meat, eggs, garlic, and onion.
16	French fries	Deep-fried potato strips. This snack is often sprinkled with salt.

Supplementary table: Description of 9 selected street foods under the desserts category in Malaysia.

**No.**	**Name of street food**	**Description of street food**
1	*Apam balik* with cheese	A sweet pancake that is filled with peanuts, sugar, corn, margarine and shredded cheese.
2	Banana fritters with cheese	Banana that is coated in a batter and deep-fried. The fritters is then topped with shredded cheese.
3	Donut	Deep-fried leavened dough, coated with caster sugar.
4	*Apam balik*	A sweet pancake that is filled with peanuts, sugar, corn, and margarine.
5	Banana fritters	Banana that is coated in a batter and deep-fried.
6	Popcorn	Corn kernels that are heated to 'pop' or expand, and coated in caramel sauce.
7	*Kuih keria*	Traditional cake made by deep-frying a mixture of mashed sweet potatoes and wheat flour. The fried cake is then coated with sugar syrup.
8	*Kuih jelurut*	A traditional cake made of a thin batter (a mixture of rice flour, sugar, salt, coconut milk) that is filled in a cone-shaped coconut leaf and steamed.
9	*Kuih seri muka*	A Malay traditional cake that comprises a layer of steamed glutinous rice and another layer of green custard made from pandan juice.
