# Supplementary material for: Fatty Acid Composition of Selected Street Foods Commonly Available in Malaysia
Source: Foods. 2023 Mar 14;12(6):1234. doi: 10.3390/foods12061234 (PMC10048182; doi:10.3390/foods12061234)
Supplement: Supplementary file 1 [file foods-12-01234-s001.zip › Table S3.pdf]

TABLE S3: MUFA CONTENT IN 39 STREET FOODS FROM HIGHEST TO LOWEST AMOUNT

**No.**	**Street Food**	**Food Category**	**Average MUFA content (%)**
1	Fried vermicelli @ fried *mihun*	Main meals	44.4 ± 1.5
2	Fried *kuey teow*	Main meals	43.0 ± 0.0
3	*Murtabak*	Snacks	43.0 ± 0.0
4	Fried rice	Main meals	43.0 ± 0.0
5	Fried chicken	Snacks	42.2 ± 1.7
6	Curry puff	Snacks	41.0 ± 2.0
7	Fried chicken with cheese	Snacks	41.0 ± 0.0
8	Banana fritters	Desserts	41.0 ± 0.0
9	*Kuih keria*	Desserts	41.0 ± 0.0
10	*Kerepek*	Snacks	40.9 ± 0.2
11	Fried noodles	Main meals	40.6 ± 0.9
12	Fried *popiah*	Snacks	40.6 ± 0.2
13	Chicken nuggets	Snacks	40.4 ± 2.8
14	Fried fish ball	Snacks	40.1 ± 0.2
15	Donut	Desserts	40.0 ± 2.8
16	Chicken rice	Main meals	39.5 ± 0.7
17	Chicken burger	Main meals	39.0 ± 0.0
18	Banana fritters with cheese	Desserts	38.3 ± 0.4
19	*Keropok lekor*	Snacks	38.3 ± 2.5
20	*Roti john*	Main meals	37.9 ± 0.2
21	*Roti canai* (flat bread)	Main meals	37.8 ± 0.0
22	*Cakoi*	Snacks	37.6 ± 5.9
23	French fries	Snacks	37.3 ± 0.4
24	Fried sausage	Snacks	37.3 ± 5.3
25	*Nasi lemak*	Main meals	34.7 ± 6.0
26	Net crepes *(Roti jala)*	Main meals	34.3 ± 1.9
27	Satay	Snacks	33.0 ± 0.0
28	Kebab	Main meals	32.7 ± 0.4
29	*Nasi lemak* with fried chicken	Main meals	32.5 ± 0.7
30	Noodles with gravy (Curry / Soy sauce)	Main meals	32.4 ± 0.2
31	Takoyaki	Snacks	32.0 ± 2.9
32	*Apam balik*	Desserts	32.0 ± 5.9
33	Popcorn	Desserts	27.5 ± 0.8
34	Fried chicken (non-meat parts)	Snacks	25.5 ± 0.7
35	*Apam balik* with cheese	Desserts	21.9 ± 1.0
36	*Kuih seri muka*	Desserts	18.5 ± 0.7
37	Pizza	Snacks	15.6 ± 1.3
38	Glutinous rice with *rendang* (meat cooked with spices)	Main meals	11.7 ± 0.5
39	*Kuih jelurut*	Desserts	7.0 ± 0.0
