# Supplementary material for: Fatty Acid Composition of Selected Street Foods Commonly Available in Malaysia
Source: Foods. 2023 Mar 14;12(6):1234. doi: 10.3390/foods12061234 (PMC10048182; doi:10.3390/foods12061234)
Supplement: Supplementary file 1 [file foods-12-01234-s001.zip › Table S4.pdf]

TABLE S4: PUFA CONTENT IN 39 STREET FOODS FROM HIGHEST TO LOWEST AMOUNT

No.	Street Food	Food Category	Average PUFA content (%)
1	*Apam balik*	Desserts	38.8 ± 8.8
2	*Apam balik* with cheese	Desserts	38.5 ± 1.2
3	Takoyaki	Snacks	34.5 ± 1.0
4	Pizza	Snacks	33.4 ± 2.1
5	Kebab	Main meals	32.0 ± 1.3
6	Fried chicken (non-meat parts)	Snacks	30.5 ± 0.7
7	Satay	Snacks	30.0 ± 0.0
8	Fried sausage	Snacks	24.6 ± 7.3
9	French fries	Snacks	22.5 ± 0.6
10	Fried chicken with cheese	Snacks	21.0 ± 0.0
11	*Roti john*	Main meals	19.0 ± 0.5
12	*Kerepek*	Snacks	18.0 ± 0.0
13	Noodles with gravy (Curry / Soy sauce)	Main meals	17.7 ± 0.2
14	Chicken burger	Main meals	17.0 ± 0.0
15	Chicken rice	Main meals	16.9 ± 1.3
16	Chicken nuggets	Snacks	16.9 ± 4.1
17	Fried noodles	Main meals	16.5 ± 3.6
18	Banana fritters with cheese	Desserts	16.4 ± 0.3
19	*Nasi lemak*	Main meals	15.8 ± 3.3
20	*Keropok lekor*	Snacks	15.5 ± 3.5
21	*Roti canai* (flat bread)	Main meals	15.2 ± 0.3
22	Fried chicken	Snacks	14.8 ± 3.7
23	Fried rice	Main meals	14.0 ± 0.0
24	*Kuih keria*	Desserts	14.0 ± 0.0
25	Popcorn	Desserts	13.1 ± 0.5
26	*Nasi lemak* with fried chicken	Main meals	13.0 ± 0.0
27	Donut	Desserts	12.8 ± 1.1
28	Fried fish ball	Snacks	12.7 ± 0.3
29	Fried vermicelli @ fried *mihun*	Main meals	12.7 ± 0.9
30	Fried *popiah*	Snacks	12.6 ± 0.7
31	Curry puff	Snacks	12.5 ± 1.8
32	Net crepes *(Roti jala)*	Main meals	12.4 ± 0.6
33	Fried *kuey teow*	Main meals	12.0 ± 0.0
34	Banana fritters	Desserts	12.0 ± 0.0
35	*Murtabak*	Snacks	11.0 ± 0.0
36	*Cakoi*	Snacks	10.1 ± 1.7
37	*Kuih seri muka*	Desserts	8.0 ± 0.0
38	Glutinous rice with *rendang* (meat cooked with spices)	Main meals	4.8 ± 1.2
39	*Kuih jelurut*	Desserts	4.5 ± 0.7
